# Thiadiazole–azetidinone sulfonamide hybrids with antimycobacterial activity supported by structure-based analysis

**DOI:** 10.1039/d6ra00735j

**Published:** 2026-03-19

**Authors:** Subham Kumar Vishwakarma, Achal Mishra, Naresh Panigrahi, Cesar Augusto Roque-Borda

**Affiliations:** a GITAM School of Pharmacy, GITAM Deemed to be University Rushikonda Visakhapatnam-530045 AP India; b School of Pharmaceutical Sciences, São Paulo State University (UNESP) 14800903 Araraquara Brazil; c Department of Pharmacy, Guru Ghasidas Vishwavidyalaya Bilaspur– 495009 CG India; d Vicerrectorado de Investigación, Universidad Católica de Santa María Arequipa Peru cesar.roque@ucsm.edu.pe

## Abstract

Tuberculosis (TB), caused by *Mycobacterium tuberculosis*, remains a major global health challenge, exacerbated by the rapid emergence of drug-resistant strains. In this study, a series of thiadiazole–azetidinone hybrid molecules was designed and synthesized by integrating two pharmacophores with known relevance in antimycobacterial drug discovery. The hybrid framework was conceived to explore the structural compatibility of thiadiazole-2-sulfonamide and azetidinone motifs within a single molecular architecture targeting two essential mycobacterial enzymes, decaprenylphosphoryl-β-d-ribose 2′-oxidase (DprE1) and dihydrofolate reductase (DHFR), involved in cell wall biosynthesis and folate metabolism, respectively. The synthesized compounds displayed *in vitro* antimycobacterial activity against *M. tuberculosis* H37Rv and were further analyzed through molecular docking and molecular dynamics simulations (200 ns) to rationalize their interactions with both targets under dynamic conditions. These computational studies provided mechanistic insights into the binding modes, stability, and key interactions governing enzyme recognition within this hybrid series. *In silico* ADMET analysis indicated acceptable drug-like profiles across the scaffold. Rather than defining a clinically optimized candidate, this work establishes a structure–activity and structure–interaction framework that supports the thiadiazole–azetidinone hybrid concept and guides future chemical optimization toward antitubercular agents.

## Abstract 2

Hybrid molecules active against *Mycobacterium tuberculosis* reveal interactions with the essential targets DprE1 and DHFR through structure-based analysis.

## Graphical abstract

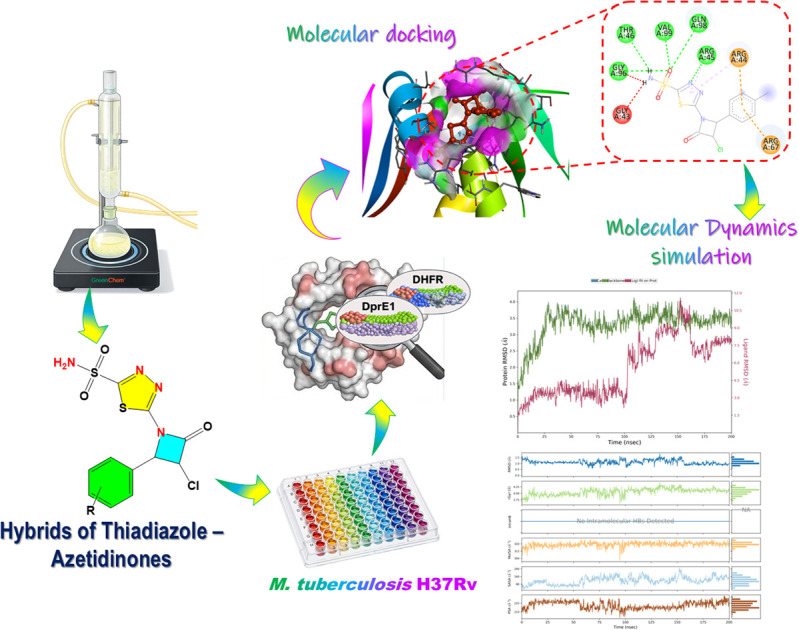

## 1. Introduction

Tuberculosis (TB), caused by *M. tuberculosis*, remains one of the leading causes of mortality from infectious diseases worldwide, with drug-resistant strains representing a persistent and escalating threat to global health. According to the World Health Organization, more than 10 million new TB cases and over one million deaths were reported in 2024, underscoring the sustained burden of the disease, particularly in low- and middle-income countries.^1–4^ Despite the long-standing availability of combination chemotherapy, prolonged treatment regimens, drug-associated toxicity, and limited patient adherence continue to drive the emergence and dissemination of multidrug-resistant (MDR) and extensively drug-resistant (XDR) *M. tuberculosis* strains.^5–7^

The current antitubercular drug pipeline has struggled to keep pace with the adaptive capacity of *M. tuberculosis*. Even recently approved agents, including bedaquiline, delamanid, and pretomanid, have already been associated with reports of emerging resistance, highlighting the fragility of the existing therapeutic arsenal and the urgent need for structurally and mechanistically distinct chemical entities.^5–7^ In this context, drug discovery strategies that prioritize novel molecular scaffolds and target essential bacterial pathways remain a central objective in tuberculosis research.

Among validated targets in *M. tuberculosis*, decaprenylphosphoryl-β-d-ribose 2′-oxidase (DprE1) and dihydrofolate reductase (DHFR) play critical and non-redundant roles in bacterial survival. DprE1 is a key enzyme involved in the biosynthesis of the arabinan domain of the mycobacterial cell wall, and its inhibition leads to severe structural defects and bacterial death.^8^ DHFR, by contrast, is essential for folate metabolism and nucleotide biosynthesis, rendering it indispensable for DNA synthesis and cellular replication.^9^ Simultaneous interference with these pathways offers a complementary approach to disrupt both cell wall integrity and metabolic homeostasis in *M. tuberculosis*.

Heterocyclic scaffolds such as sulfonamides, azetidinones, and thiadiazole derivatives have been explored for antimicrobial drug discovery. Sulfonamide derivatives disrupt key folate-dependent metabolic pathways by targeting enzymes such as dihydropteroate synthase and dihydrofolate reductase, which are essential for nucleotide biosynthesis in *M. tuberculosis* and other bacteria.^10^ Azetidinone frameworks, as β-lactam analogs, have been associated with antibacterial activity related to interference with cell wall biosynthesis and structural integrity.^11^ 1,3,4-Thiadiazole-containing compounds have attracted attention due to their structural versatility, favorable physicochemical profiles, and broad antimicrobial activity *in vitro*.^12^ Together, these scaffolds provide a rational basis for hybridization aimed at simultaneously perturbing multiple essential pathways in mycobacterial physiology.^13^

The present study focuses on the design, synthesis, and evaluation of thiadiazole-2-sulfonamide–azetidinone hybrid compounds as a means of assessing scaffold compatibility and antimycobacterial relevance rather than immediate potency optimization. Biological evaluation was complemented by molecular docking and molecular dynamics (MD) simulations against DHFR and DprE1 to rationalize binding modes, interaction patterns, and structural stability under dynamic conditions. By integrating experimental screening with *in silico*-based analysis, this work establishes a structure–activity and structure–interaction framework intended to support further chemical refinement of thiadiazole–azetidinone hybrids as antitubercular agents.

Computational methods such as molecular docking and MD simulations are widely recognized as complementary tools in drug discovery, providing structural and mechanistic insights that support and rationalize experimental results.^14^ Molecular docking predicts ligand binding modes and relative interaction patterns, while MD simulations explore the dynamic behavior of protein–ligand complexes, accounting for conformational flexibility and stability under physiologically relevant conditions, thereby enhancing the interpretation of *in vitro* findings.^15^ Together, these *in silico* approaches contribute to understanding structure–activity relationships and guide subsequent optimization strategies in medicinal chemistry projects, reducing cost and time in early-stage candidate evaluation.^16,17^

## 2. Results and discussions

### 2.1 Antibacterial evaluation of thiadiazole–azetidinone hybrids

The antibacterial activity of the synthesized thiadiazole–azetidinone hybrids was evaluated against representative Gram-positive (*S. aureus* and *B. subtilis*) and Gram-negative (*E. coli* and *P. aeruginosa*) bacterial strains. All MIC assays were performed in triplicate, and the results were consistent across the independent experiments. Overall, the compound series exhibited measurable antibacterial activity across both bacterial classes, with several derivatives displaying inhibition levels comparable to the reference drug amoxicillin (Table 1), indicating that the hybrid scaffold is compatible with antibacterial activity against diverse bacterial targets.

**Table 1:** Minimum inhibitory concentration of newly synthesized thiadiazole sulfonamide-2-azetidinone hybrid derivatives

Samples	MIC_90_ (µg mL^−1^)			
	*E. coli*	*Pseudomonas aeruginosa*	*Staphylococcus aureus*	*Bacillus subtilis*
AZTDS-1	6.25	6.25	3.125	6.25
AZTDS-2	12.5	12.5	25	25
AZTDS-3	25	50	25	25
AZTDS-4	25	50	25	50
AZTDS-5	25	25	12.5	12.5
AZTDS-8	12.5	12.5	25	12.5
AZTDS-12	25	12.5	50	50
AZTDS-14	25	25	25	25
Amoxicillin	6.25	3.125	3.125	1.56

Within the series, AZTDS-1, AZTDS-2, and AZTDS-8 emerged as the most active derivatives, particularly against Gram-positive organisms. These compounds displayed MIC values in the low micromolar range (3.125–25 µg mL^−1^), with AZTDS-1 showing the highest potency against *S. aureus* (MIC = 3.125 µg mL^−1^), approaching the activity of the reference antibiotic. The enhanced activity observed against Gram-positive bacteria may be associated with the relatively simpler cell envelope architecture of these organisms, which can facilitate access of the thiadiazole–azetidinone framework to intracellular or membrane-associated targets.^18^

Notably, the same compounds retained moderate antibacterial activity against Gram-negative strains, with MIC values ranging from 6.25 to 25 µg mL^−1^. This observation suggests that the hybrid scaffold is not exclusively restricted to Gram-positive bacteria and may partially overcome the permeability barriers typically associated with the outer membrane of Gram-negative organisms. However, the generally reduced potency observed against Gram-negative strains compared to Gram-positive counterparts is consistent with the increased structural complexity and efflux mechanisms characteristic of these bacteria.

AZTDS-14 exhibited a distinct antibacterial profile, characterized by uniform MIC values (25 µg mL^−1^) across all tested strains. While less potent than the leading derivatives, this consistent activity suggests a broader, albeit weaker, antibacterial spectrum that may reflect differences in aryl substitution influencing physicochemical properties such as lipophilicity or membrane interaction. In contrast, the remaining derivatives displayed variable antibacterial effects, with MIC values predominantly in the moderate activity range (25–50 µg mL^−1^), highlighting the sensitivity of antibacterial performance to structural modifications within the aryl moiety. The incorporation of the azetidinone ring into the thiadiazole–sulfonamide core confers broad-spectrum antibacterial activity, while aryl substitution plays a modulatory role in determining potency and bacterial selectivity. Although no definitive structure–activity relationships can be established at this stage, the observed trends provide a preliminary framework to guide further chemical optimization aimed at enhancing antibacterial efficacy, particularly against Gram-negative pathogens.

### 2.2 Antimycobacterial activity

The antimycobacterial activity of the synthesized thiadiazole–azetidinone hybrids was evaluated against *M. tuberculosis* H37Rv. The MIC values, obtained from triplicate experiments, are summarized in Table 2. Overall, the compound series displayed reproducible inhibitory activity in the low tens of micrograms per milliliter range, indicating that the hybrid scaffold is compatible with growth inhibition of *M. tuberculosis* under *in vitro* conditions. Within the series, AZTDS-1 emerged as the most active derivative, exhibiting an MIC value of 12.5 µg mL^−1^, whereas the remaining analogues showed MIC values clustered around 25 µg mL^−1^. Although this level of activity remains modest compared to first-line antitubercular agents, it is consistent with early-stage hit compounds targeting intracellular enzymes of *M. tuberculosis*, where limited permeability, active efflux, and metabolic adaptation frequently constrain apparent *in vitro* potency.^19^

**Table 2:** Antimycobacterial activity (MIC) of compounds against strains of *M. tuberculosis*

Test compound	*M. tuberculosis* H37Rv (µg mL^−1^)
AZTDS-1	12.5
AZTDS-2	25
AZTDS-3	25
AZTDS-4	25
AZTDS-5	25
AZTDS-8	25
AZTDS-12	25
AZTDS-14	25
RIFAMPICIN	0.78
*p*-Amino salicylic acid	1.56

The narrow distribution of MIC values across most derivatives suggests that the thiadiazole–azetidinone core contributes a baseline antimycobacterial effect, while aryl substitution at the azetidinone moiety modulates activity rather than driving dramatic potency shifts. The superior performance of AZTDS-1 relative to its analogues indicates that specific substituent-dependent interactions may enhance target engagement or cellular accessibility, an effect commonly observed during early optimization of antitubercular scaffolds.^20^ Related thiadiazole–azetidinone frameworks have previously shown antimycobacterial activity in the low micromolar to single-digit µg mL^−1^ range, where incorporation of the azetidinone ring was proposed to increase hydrophobicity and facilitate penetration through the mycobacterial cell envelope.^21^ The comparable activity of AZTDS-1 suggests that the present hybrid scaffold preserves this structural feature while allowing substituent-dependent modulation of target engagement. In contrast, rifampicin and *p*-aminosalicylic acid exhibited substantially lower MIC values under identical experimental conditions, reflecting their optimized pharmacological profiles and validated mechanisms of action. The large potency gap between these reference drugs and the present compounds underscores that the latter should not be considered clinical leads at this stage, but rather chemically tractable starting points for further refinement.

Given the essential roles of DprE1 in arabinan biosynthesis and DHFR in folate-dependent nucleotide metabolism, both of which are well-established vulnerabilities in *M. tuberculosis*,^22,23^ the observed antimycobacterial activity prompted an exploration of potential molecular interactions with these targets. Subsequent *in silico* studies were therefore undertaken to rationalize activity differences within the series and to identify structural features that could guide future optimization, rather than to assign definitive mechanisms of action.

### 2.3 Docking-based evaluation of AZTDS binding to essential mycobacterial enzymes

Molecular docking was employed as a structure-based approach to explore the compatibility of the synthesized thiadiazole–azetidinone hybrids with two validated mycobacterial targets, DHFR and DprE1. Docking calculations are widely used in early drug discovery to rationalize ligand–target recognition and to prioritize compounds for further investigation, particularly when integrated with experimental screening data.^24,25^

**Fig. 1:**
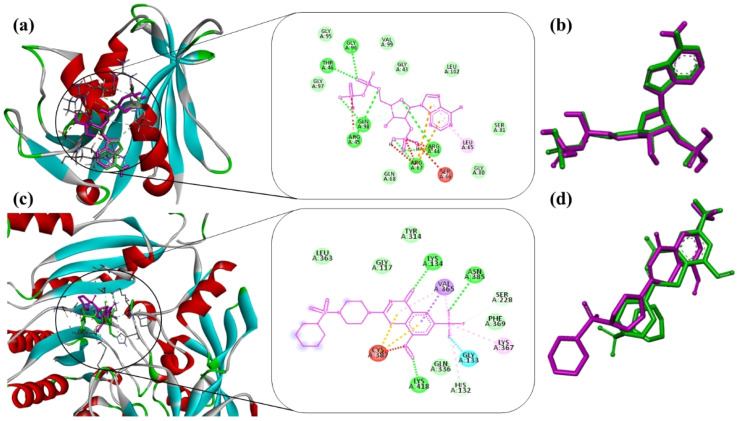
Validation of docking; (a) co-crystalized ligand (Pink Stick) interaction with the DHFR protein active site with their 2D interaction; (b) superimposed pose of the co-crystallized inhibitor of DHFR protein (PDB ID: 4KLX) for the validation of docking protocol; (c) co-crystalized ligand (Pink Stick) interaction with the DprE1 protein active site with their 2D interaction; (d) superimposed pose of the co-crystallized inhibitor of DprE1 protein (PDB ID: 6G83) for the validation of docking protocol.

Prior to docking the compound series, the reliability of the docking protocol was assessed by redocking the co-crystallized ligands into the active sites of DHFR (PDB ID: 4KLX) and DprE1 (PDB ID: 6G83). The redocked poses closely reproduced the experimentally observed binding conformations, yielding RMSD values of 0.00 Å for DHFR and 0.04 Å for DprE1. RMSD values below 2 Å are generally considered indicative of a valid docking setup capable of reproducing native ligand geometries,^25^ supporting the suitability of the protocol for comparative binding analysis (Fig. 1).

Following validation, nine AZTDS derivatives were subjected to docking studies based on their observed antimycobacterial activity. DHFR was selected as a representative metabolic target due to its essential role in folate metabolism and DNA biosynthesis in *M. tuberculosis*, as well as its long-standing validation as an antitubercular drug target.^26^ Among the tested compounds, AZTDS-2 exhibited the most favorable predicted binding affinity toward DHFR (−8.5 kcal mol^−1^), forming multiple hydrogen bonds with key active-site residues including Arg44, Arg45, Thr46, Gly96, Gln98, and Val99. These residues have been previously implicated in ligand recognition and catalytic stabilization within the mycobacterial DHFR active site.^27^

DprE1 was selected as a second target due to its indispensable role in arabinan biosynthesis and cell wall assembly in *M. tuberculosis*, and because inhibition of DprE1 has been shown to result in rapid bactericidal effects.^22^ More specifically, DprE1 is a flavin adenine dinucleotide (FAD)-dependent oxidoreductase that catalyzes the oxidation of decaprenylphosphoryl-β-d-ribose (DPR) to decaprenylphosphoryl-β-d-2′-keto-*erythro*-pentafuranose (DPX), which is subsequently converted into decaprenylphosphoryl-β-d-arabinose (DPA), the essential arabinose donor required for the assembly of arabinogalactan and lipoarabinomannan. Because DprE1 has also been genetically validated and is located in an extracytoplasmic environment, it has emerged as one of the most tractable and pharmacologically relevant antitubercular targets. Consistent with this biological relevance, inhibition of DprE1 has been associated with rapid disruption of cell wall homeostasis and potent bactericidal effects. In line with this view, our docking results suggest structural compatibility of AZTDS derivatives with the catalytic cavity of DprE1, as the predicted binding poses occupy regions analogous to those reported for experimentally validated DprE1 inhibitor series that induce rapid growth arrest and cell lysis in mycobacteria.^28^

Docking analysis indicated that AZTDS-14 achieved the highest predicted affinity for DprE1 (−8.2 kcal mol^−1^), establishing hydrogen-bond interactions with residues such as His132, Gly133, Gln336, Asn385, and Cys387. This interaction pattern appears consistent with those reported for recently described piperazinyl–*N*-arylacetamide/1,3,4-thiadiazole and quinoxaline–thiadiazole scaffolds, in which key contacts with His132 and Cys387, together with π-mediated interactions around the substrate-binding domain, were shown to underpin stable binding modes within the DprE1 active site.^29^ Notably, Cys387 has been identified as a critical residue involved in the binding and inhibition of several clinically relevant DprE1 inhibitors,^30^ suggesting that AZTDS-14 occupies a chemically relevant region of the active site. Moreover, covalent and non-covalent DprE1 inhibitors that retain productive engagement of Cys387 have demonstrated tight correlation between biochemical potency, target engagement in overexpression/MC-suppression systems, and whole-cell activity, suggesting that AZTDS-14 may occupy a chemically relevant region of the catalytic pocket rather than representing an artefactual docking orientation.^28^AZTDS-1, which displayed the most favorable antimycobacterial activity *in vitro*, demonstrated balanced interaction profiles against both targets. In DHFR, AZTDS-1 showed a predicted binding affinity of −7.3 kcal mol^−1^ and formed hydrogen bonds with Arg45, Thr46, Gly96, Gly97, and Val99. In addition, hydrophobic and π-mediated interactions involving Arg45 and Ala126 contributed to stabilization of the ligand within the binding pocket. Such mixed polar and hydrophobic interaction patterns are characteristic of effective DHFR inhibitors.^27^ Notably, recent docking-guided optimization of thiadiazole–thiazolidinone–quinoxaline hybrids against DHFR also relied on maximizing this combination of hydrogen bonds in the conserved ‘arginine clamp’ and hydrophobic packing deeper in the cavity to prioritize compounds for synthesis and antimycobacterial testing, underscoring the relevance of the interaction pattern observed for AZTDS-1 and AZTDS-2.^31^

**Fig. 2:**
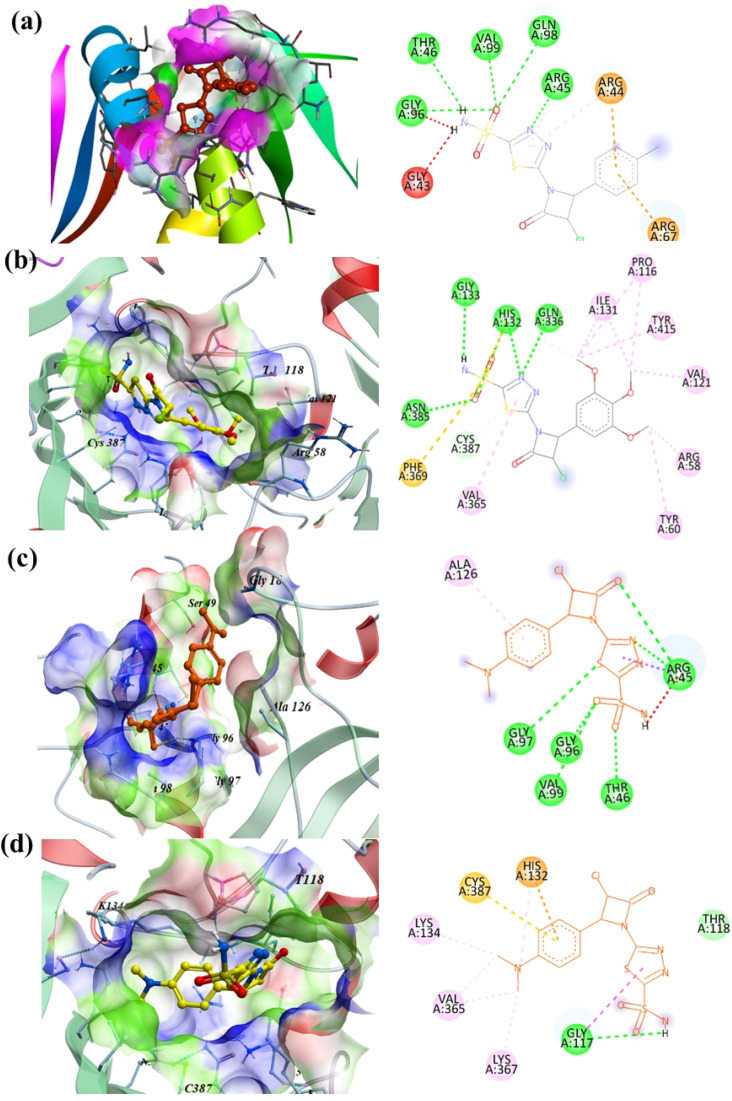
Visualization of two-dimensional and three-dimensional docking interaction poses: (a) binding of AZTDS-2 with DHFR (PDB: 4KLX); (b) binding of AZTDS-14 with DprE1 (PDB: 6G83); (c) binding of AZTDS-1 with 4KLX; (d) binding of AZTDS-1 with DprE1.

Docking against DprE1 further revealed that AZTDS-1 is capable of engaging this enzyme through a combination of hydrogen bonding and hydrophobic contacts. A hydrogen bond with Gly117 was observed, along with stabilizing interactions involving His132, Val135, Lys134, and Lys367. Comparable networks of hydrogen bonds and hydrophobic contacts involving Gly117, His132, Lys134 and neighboring residues have been observed for other noncovalent DprE1 inhibitors, including thiadiazole-based hybrids and quinoxaline derivatives, where such mixed polar–hydrophobic interaction profiles were further validated by long-timescale molecular dynamics as compatible with stable binding in the catalytic cleft.^29^ These residues are located within or adjacent to the catalytic cavity and have been reported to participate in ligand recognition and stabilization in structural studies of DprE1–inhibitor complexes.^22,30^ Taken together, the docking poses of AZTDS-1 and AZTDS-14 suggest that our thiadiazole–azetidinone framework recapitulates multiple ‘hot spot’ contacts that have emerged as recurrent features among chemically diverse DprE1 inhibitors, suggesting that DprE1 may represent a plausible functional target for this series.^28^ Representative two- and three-dimensional interaction poses of AZTDS-1, AZTDS-2, and AZTDS-14 within DHFR and DprE1 are illustrated in Fig. 2.

Although several compounds exhibited favorable docking scores, no direct one-to-one correlation between predicted binding affinity and antimycobacterial potency was observed. This observation aligns with previous DprE1-targeted discovery campaigns, where compound series with nanomolar enzymatic inhibition and clear genetic/biophysical evidence of DprE1 engagement still displayed a wide spread of MIC values, largely driven by solubility, lipophilicity, efflux susceptibility and the notoriously variable permeability of the mycobacterial cell envelope.^28,29,31^ This lack of direct correlation is consistent with previous studies showing that cellular activity against *M. tuberculosis* is influenced by multiple factors beyond target binding, including cell wall permeability, efflux mechanisms, and intracellular compound accumulation.^19^ Comparable behavior has been reported for thiadiazole-based hybrids against both DprE1 and DHFR, where compounds prioritized solely on the basis of docking score often required additional optimization of physicochemical properties (*e.g.*, log *P*, polar surface area, rotatable bonds) before achieving MIC values comparable to first-line drugs in the microplate.^29,31^ The comparative docking scores of all AZTDS derivatives against both targets are summarized in Table 3.

**Table 3:** Molecular docking score of synthesized compounds (AZTDS) with target proteins: DHFR (PDB: 4KLX) and DprE1 (PDB: 6G83)

Sample	Molecular docking score (kcal mol^−1^)	
	DHFR (PDB: 4KLX)	DprE1 (PDB: 6G83)
AZTDS-1	−7.3	−7.5
AZTDS-2	−8.5	−8.0
AZTDS-3	−7.0	−7.2
AZTDS-4	−7.1	−7.7
AZTDS-5	−7.1	−7.4
AZTDS-8	−7.0	−7.7
AZTDS-12	−6.1	−7.6
AZTDS-14	−7.3	−8.2

Overall, the docking analysis suggests the structural feasibility of the thiadiazole–azetidinone scaffold to interact with both DHFR and DprE1. Structure–activity relationship (SAR) considerations further provide insight into the molecular features contributing to the activity of AZTDS-1. AZTDS-1 is a hybrid scaffold composed of a thiadiazole core, an azetidinone ring, and a *para*-dimethylaminophenyl substituent, each contributing to the interaction profile observed within the active sites of both enzymes. The thiadiazole moiety functions as the central pharmacophore, facilitating recognition within the catalytic cavities of DHFR and DprE1 through polar interactions and hydrogen-bonding contacts with residues lining the active site. This heterocyclic core is well positioned to establish interactions with conserved residues involved in substrate stabilization, thereby contributing to ligand anchoring within the binding pocket. Similar arrangements of polar contacts with the Arg44/Arg45–Thr46–Gly96 region, complemented by hydrophobic and π-stacking interactions toward the back of the pocket, have been described for other thiadiazole-containing and quinoxaline-based DHFR inhibitors, and are typically associated with retention of activity across bacterial DHFR isoforms.^31^ The azetidinone ring provides a conformationally constrained scaffold that maintains the spatial orientation of the substituents relative to the heterocyclic core. This rigidity favors optimal positioning of the ligand within the binding cavity and supports stable interactions with residues surrounding the catalytic pocket of both targets. Structural rigidity introduced by the azetidinone moiety is therefore likely to contribute to the overall stabilization of the ligand–protein complex.

The *para*-dimethylaminophenyl substituent plays a critical role in modulating the electronic and steric properties of the molecule. The electron-donating dimethylamino group (–N(CH_3_)_2_) increases electron density across the aromatic system, enhancing the capacity of the ligand to participate in hydrogen bonding and electrostatic interactions with residues within the active site. In addition, the substituted phenyl ring contributes to hydrophobic packing within nonpolar regions of the enzyme cavities. Such steric and hydrophobic contributions allow the ligand to occupy complementary pockets within the binding site and promote tighter packing against surrounding residues. Consistent with the docking results, the dimethylaminophenyl moiety contributes to several key interactions within DHFR. Hydrogen-bond contacts were observed with residues including Gly96, Thr46, Val99, and Arg45, while the aromatic ring enables π–π stacking interactions with Tyr60, further stabilizing the ligand within the catalytic pocket. Similar interaction patterns were also observed in DprE1, where the substituent participates in hydrogen bonding with residues such as Gly117, His132, and Lys134, while additional hydrophobic contacts with residues including Trp16 and Tyr60 contribute to stabilization within the enzyme cavity.

Together, these interaction features suggest that the dimethylaminophenyl group plays a central role in promoting favorable binding interactions with both targets. Notably, the presence of the *para*-dimethylaminophenyl substituent distinguishes AZTDS-1 from the other derivatives and appears to play a decisive role in its improved antimycobacterial activity. The electron-donating character and steric bulk of this group enable additional hydrogen-bonding and π-mediated interactions within the DHFR and DprE1 binding pockets, as observed in the docking analysis. These interactions may provide a structural rationale for the improved biological activity observed for AZTDS-1. Based on the combined docking analysis and SAR considerations, AZTDS-1 was selected for subsequent molecular dynamics simulations to evaluate the temporal stability of the predicted binding modes and to further refine the structural hypotheses governing ligand–target recognition.^15^

### 2.4 Molecular dynamics simulation

MD simulations were performed to evaluate the temporal stability and dynamic behavior of the AZTDS-1 complexes with DHFR and DprE1. Each protein–ligand system was simulated for 200 ns using the Desmond simulation package. Trajectory analyses included RMSD, RMSF, radius of gyration (RoG), and detailed protein–ligand interaction profiling, providing insight into conformational stability and interaction persistence throughout the simulations. RMSD analysis was first used to assess the overall structural stability of the complexes. For the DHFR–AZTDS-1 system, the protein backbone RMSD stabilized between approximately 2.5 and 3.5 Å after an initial equilibration period of ∼50 ns. Although moderate fluctuations were observed, these remained within the expected range for a protein of this size and did not indicate large-scale structural rearrangements. The persistence of RMSD values within this range suggests that the system reached equilibrium and maintained a stable conformational state for the remainder of the simulation.

In the case of the DprE1–AZTDS-1 complex, backbone RMSD values remained below 3 Å throughout the trajectory, indicating a comparatively more rigid system. Equilibration was achieved within the first ∼25 ns, after which the RMSD plateaued, reflecting stable protein architecture. Analysis of Cα RMSD showed tighter convergence (approximately 1.5–2.5 Å), supporting preservation of the protein's structural integrity. Importantly, the ligand heavy-atom RMSD remained below 3 Å across the full simulation, indicating sustained binding without evidence of dissociation or major reorientation within the active site. RMSF analysis was conducted to examine residue-level flexibility and to identify regions contributing to local motion. For the DHFR–AZTDS-1 complex, RMSF values ranged from approximately 0.70 to 3.66 Å for most residues. Elevated fluctuations were observed for surface-exposed and loop regions, including residues such as Pro316 and Trp6, which are distal from the binding pocket and typically exhibit higher flexibility. Residues lining the ligand-binding site displayed comparatively lower RMSF values, suggesting that ligand association contributes to local stabilization.

**Fig. 3:**
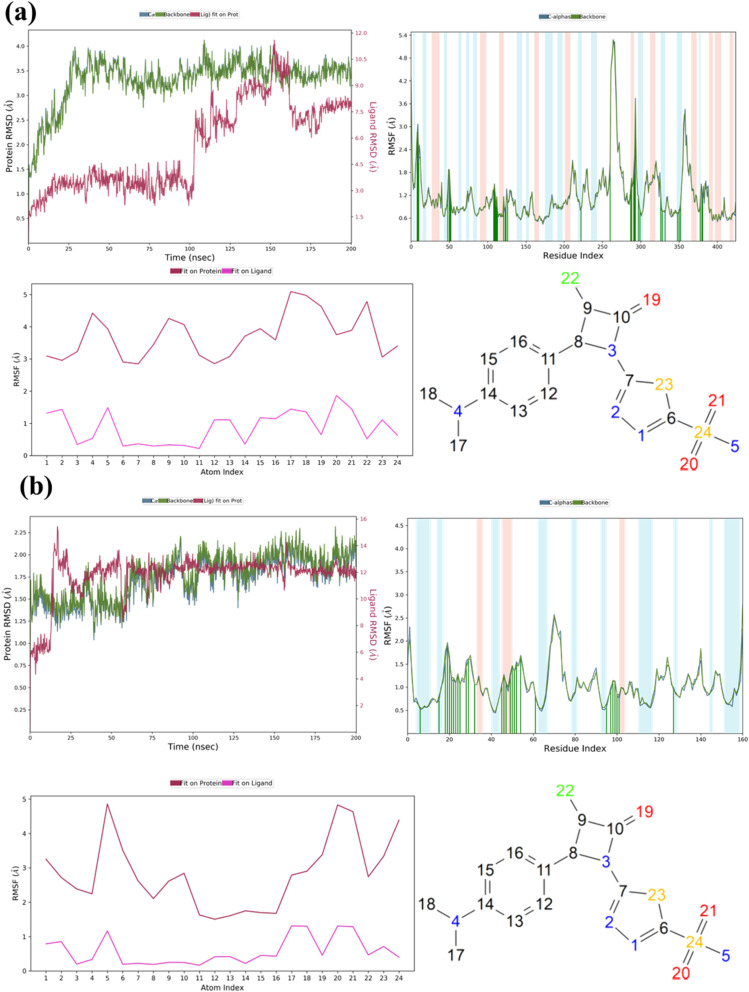
Root Mean Square Deviation and Root Mean Square Fluctuation of most active protein–ligand complexes. (a) 4KLX with AZTDS-1, (b) DprE1 with AZTDS-1.

In the DprE1–AZTDS-1 complex, RMSF values were generally lower, with most residues fluctuating within 0.5–2.0 Å. Regions displaying increased flexibility were located in loop segments distant from the catalytic cavity, notably around residues 240–270 and 320–340. Importantly, residues directly involved in ligand recognition, including Gly117, His132, Val135, and Lys134, exhibited limited fluctuations, indicating a rigid and well-defined binding environment. The RMSD and RMSF analyses for both complexes are summarized in Fig. 3.

Protein–ligand interaction analysis provided additional insight into the stabilization of AZTDS-1 within both binding sites. In the DHFR–AZTDS-1 complex, persistent hydrogen bonds were observed with residues including Gly117, Lys418, and His132, contributing significantly to ligand stabilization. Among these, the interaction involving Gly117 was particularly dominant, accounting for approximately 43% of the interaction occupancy. Additional stabilization arose from ionic contacts with Arg58 and Asp389, as well as π–π stacking interactions between AZTDS-1 and Tyr60. Water-mediated hydrogen bonds further reinforced ligand retention, contributing approximately 9% of the total interaction profile.

**Fig. 4:**
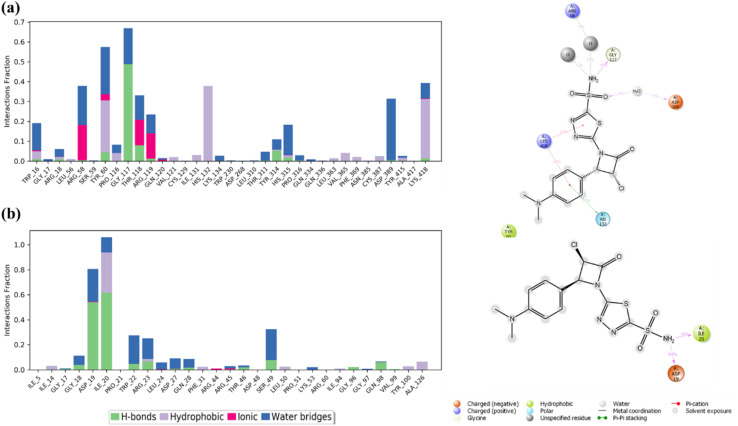
Ligand level analysis showing contribution of hydrogen bonds, hydrophobic interaction, ionic bonds, and water bridges. Residue level interaction high lightning functional groups involves in key interaction of (a) DHFR with AZTDS-1 (b) DprE1 with AZTDS-1.

In the DprE1–AZTDS-1 complex, stable hydrogen bonds were primarily formed with residues such as Gly17, Tyr60, and Lys418, which remained engaged for a substantial fraction of the simulation. Hydrophobic contacts played a complementary role, with the aromatic moieties of AZTDS-1 participating in π–π stacking interactions involving Trp16 and Tyr60, collectively supporting a well-maintained binding geometry over the full simulation timeframe. A global analysis of protein–ligand contacts along the MD trajectories indicates that AZTDS-1 is stabilized by a balanced combination of hydrogen bonds, hydrophobic interactions, ionic contacts, and water bridges in both systems, with their relative contributions summarized in Fig. 4. In the DHFR complex, the interaction network is characterized by transient hydrogen bonds and adaptable hydrophobic contacts that reorganize during the simulation without leading to ligand dissociation, consistent with a flexible binding environment.

In contrast, the DprE1–AZTDS-1 complex displays a more persistent interaction network, reflected in lower ligand RMSD fluctuations and reduced variability of key residue contacts (Fig. 3 and 4), indicative of a more constrained binding mode. Overall, the MD simulations demonstrate that AZTDS-1 remains stably bound to both targets over the simulated timescale, while revealing distinct dynamic behaviors between the two complexes. The more persistent interaction profile observed for DprE1 provides a structural rationale for the docking results and supports its consideration in subsequent structure-based optimization, without implying definitive target selectivity at this stage.

Throughout the simulations, the structural integrity of both DHFR and DprE1 was further assessed through secondary structure analysis. The presence and retention of key secondary structural elements (SSEs) indicate that ligand binding did not induce destabilizing conformational changes in either protein. DHFR and DprE1 exhibited well-preserved α-helical contents of 19.73% and 10.48%, respectively, along with β-strand contributions of 20.83% and 29.42%, confirming the maintenance of their native fold during the simulations. Ligand-centered analyses further supported the stability of the complexes. In both the DHFR–AZTDS-1 and DprE1–AZTDS-1 systems, the ligand maintained a stable conformation throughout the trajectory. The radius of gyration (RoG) remained essentially constant, indicating minimal changes in ligand compactness and overall shape during binding. Notably, the absence of persistent intramolecular hydrogen bonds within AZTDS-1 suggests that ligand stability is predominantly governed by protein-mediated interactions rather than internal self-stabilization.

**Fig. 5:**
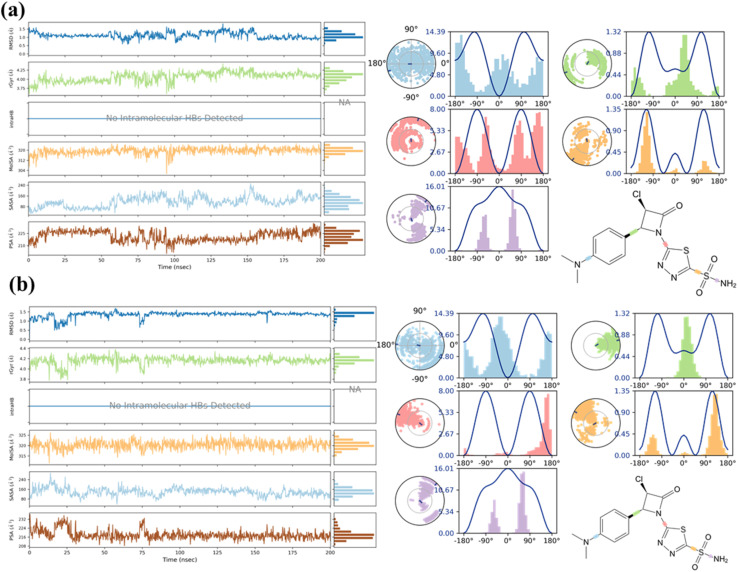
Time evaluation plot of ligand RMSD, radius of gyration (rGyr), molecular surface area (MolSA), solvent accessibility surface area (SASA), polar surface area (PSA), and ligand torsions. (a) DHFR with AZTDS-1 and, (b) DprE1 with AZTDS-1.

Analysis of molecular surface descriptors revealed moderate fluctuations in both molecular surface area (MolSA) and solvent-accessible surface area (SASA), consistent with dynamic but stable interactions between the ligand, the binding pocket, and surrounding solvent molecules. In contrast, the polar surface area remained largely constant in both complexes, indicating sustained polar interactions with key residues within the binding sites. Torsional angle analysis showed no evidence of excessive strain, supporting a favorable and conformationally stable binding orientation of AZTDS-1 in both targets. Collectively, these results indicate that AZTDS-1 engages DHFR and DprE1 without perturbing protein structural integrity and maintains a stable bound conformation throughout the simulation. The combined preservation of secondary structure, ligand compactness, and surface interaction profiles supports the overall stability of both complexes and is summarized in Fig. 5.

### 2.5 MM-GBSA binding free energy analysis

The binding free energies of AZTDS-1 in complex with DHFR and DprE1 were estimated using the Molecular Mechanics–Generalized Born Surface Area (MM-GBSA) approach implemented in the Prime module of the Schrödinger suite. This post-MD analysis was performed to quantify the energetic stability of the ligand within the protein binding sites and to complement the structural insights obtained from docking and MD simulations. The calculated binding free energy (Δ*G*_bind) values and their individual energetic contributions are summarized in Table 4.

**Table 4:** Binding free energy decomposition analysis (in kcal mol^−1^) of selected protein–ligand complexes using MM-GBSA analysis

	Binding free energy (Δ*G*, kcal mol^−1^)					
	Bind	Coulomb	Covalent	Van der Waals	H-bond	Lipophilic
DHFR with AZTDS-1	−75.1651	−20.1462	−4.9916	−50.1975	−1.9578	−29.7624
DprE1 with AZTDS-1	−75.253	−19.9648	−5.017	−50.0086	−2.0222	−29.8669

AZTDS-1 exhibited comparable and favorable binding free energies toward both targets, with Δ*G*_bind values of −75.165 kcal mol^−1^ for the DHFR complex and −75.235 kcal mol^−1^ for the DprE1 complex. These values indicate energetically stable ligand–protein associations and are consistent with the sustained interactions observed during the MD trajectories. In both complexes, van der Waals interactions represented the dominant stabilizing contribution, ranging from approximately −50.0 to −50.2 kcal mol^−1^, reflecting favorable steric complementarity between AZTDS-1 and the binding pockets. Lipophilic contributions also played a substantial role, contributing between −29.76 and −29.86 kcal mol^−1^, underscoring the importance of hydrophobic interactions in ligand stabilization.

Electrostatic (Coulombic) interactions provided an additional favorable contribution, with values ranging from −19.96 to −20.15 kcal mol^−1^, supporting the involvement of polar contacts and charge-assisted interactions within the active sites. In contrast, hydrogen-bonding and covalent interaction terms contributed more modestly to the overall binding free energy, with values between −1.96 and −2.02 kcal mol^−1^ and −4.99 and −5.07 kcal mol^−1^, respectively. Although smaller in magnitude, these interactions likely act cooperatively to reinforce ligand positioning and binding specificity.

The MM-GBSA results are in good agreement with the DM analyses, indicating that AZTDS-1 forms energetically stable complexes with both DHFR and DprE1. The predominance of van der Waals and lipophilic contributions highlights the central role of hydrophobic packing and steric compatibility in ligand recognition, while electrostatic and hydrogen-bond interactions provide complementary stabilization. Together, these findings support the dynamic stability of AZTDS-1 within both protein targets and further validate its suitability for structure-based optimization efforts.

### 2.6 *In silico* ADMET predictions

*In silico* ADMET profiling was performed for the synthesized AZTDS derivatives using the SwissADME platform and the ProTox-II server. The ADME evaluation focused on key physicochemical and pharmacokinetic descriptors relevant to oral drug-likeness, including molecular weight (<500 Da), number of hydrogen-bond donors (<5), number of hydrogen-bond acceptors (<10), octanol/water partition coefficient (log *P* < 5), number of rotatable bonds (<15), and predicted aqueous solubility (*QP* log *S* range −6.5 to 0.5). Overall, the analyzed compounds complied with the accepted thresholds for drug-like behavior, indicating favorable physicochemical profiles suitable for further development. A summary of the ADME parameters is provided in Table 5.

**Table 5:** Physicochemical properties of the designed compounds (AZTDS) by SwissADME^a^

Compound code	MW	Heavy atoms	Aromatic heavy atoms	Rotatable bonds	HBA	HBD	Molar refractivity	TPSA	Log *P*	Lipinski rule violations
AZTDS-1	387.02	22	17	5	4	0	91.84	114.41	3.24	0
AZTDS-2	358.82	22	11	3	6	1	85.19	142.87	1.64	0
AZTDS-3	423.69	22	11	3	6	1	87.92	142.87	1.11	0
AZTDS-4	423.69	22	11	3	6	1	87.92	142.87	1.42	0
AZTDS-5	423.69	22	11	3	6	1	87.92	142.87	1.25	0
AZTDS-8	362.79	22	11	3	7	1	80.18	142.87	1.16	0
AZTDS-12	469.72	25	11	4	8	2	96.44	172.33	1.39	0
AZTDS-14	434.88	27	11	6	9	1	99.7	170.56	1.43	0

a MW-Molecular weight; HBD-Hydrogen Bond donor; HBA-Hydrogen Bond acceptor; TPSA-Total Polar surface area.

Predicted toxicity profiles were assessed using ProTox-II, evaluating potential hepatotoxicity, carcinogenicity, immunotoxicity, mutagenicity, and cytotoxicity. The analysis indicated that compounds AZTDS-1 to AZTDS-2, AZTDS-6 to AZTDS-11, and AZTDS-13 to AZTDS-14 were predicted to be inactive across all evaluated toxicological endpoints. In contrast, AZTDS-3, AZTDS-4, AZTDS-5, and AZTDS-15 showed predicted cytotoxicity, while AZTDS-12 was associated with both hepatotoxic and immunotoxic liabilities. The unfavorable toxicity profile of AZTDS-12 is likely related to the presence of bromine-substituted and pyridine-containing toxicophoric motifs on the phenyl ring directly linked to the azetidinone core. The predicted toxicological properties are summarized in Table 6.

**Table 6:** *In silico* toxicity analysis of the synthesized compounds using Protox 3.0 tool

Compounds code	Predicted toxicity				
	Hepatotoxicity	Carcinogenicity	Immunotoxicity	Mutagenicity	Cytotoxicity
AZTDS-1	Inactive	Inactive	Inactive	Inactive	Inactive
AZTDS-2	Inactive	Inactive	Inactive	Inactive	Inactive
AZTDS-3	Inactive	Inactive	Inactive	Inactive	**Active**
AZTDS-4	Inactive	Inactive	Inactive	Inactive	**Active**
AZTDS-5	Inactive	Inactive	Inactive	Inactive	**Active**
AZTDS-8	Inactive	Inactive	Inactive	Inactive	Inactive
AZTDS-12	**Active**	Inactive	**Active**	Inactive	Inactive
AZTDS-14	Inactive	Inactive	Inactive	Inactive	Inactive

## 3. Study limitations and conclusions

This study describes the design, synthesis, biological evaluation, and computational analysis of a series of AZTDS as antibacterial and antimycobacterial agents. By integrating experimental screening with structure-based modeling, the work provides a coherent framework to rationalize activity trends while delineating the strengths and limitations of the proposed scaffold. From a chemical perspective, the modular combination of a 1,3,4-thiadiazole sulfonamide core with an azetidinone moiety enabled systematic exploration of aryl substitution effects without disrupting synthetic accessibility. The resulting compounds exhibited consistent physicochemical properties compatible with drug-like space, as supported by *in silico* ADMET predictions. Importantly, the synthetic strategy allowed direct comparison across derivatives, facilitating structure–activity interpretation.

Biological evaluation revealed that the AZTDS series displays broad-spectrum antibacterial activity against both Gram-positive and Gram-negative bacteria, with selected derivatives achieving MIC values comparable to the reference drug amoxicillin. Notably, several compounds retained activity across both bacterial classes, indicating that incorporation of the azetidinone unit does not compromise permeability across distinct bacterial envelopes. While activity levels varied with aryl substitution, the data collectively demonstrate that the thiadiazole–azetidinone framework is intrinsically compatible with antibacterial efficacy. Extension of the screening to *M. tuberculosis* H37Rv showed moderate but reproducible antimycobacterial activity across the compound series. AZTDS-1 emerged as the most active derivative, while the remaining compounds displayed uniform but weaker inhibition. Although these MIC values remain inferior to those of first-line antitubercular agents, they establish the scaffold as biologically competent against mycobacteria and suitable for further optimization rather than as a finished therapeutic solution.

To rationalize these activity trends at the molecular level, docking and MD simulations were employed as hypothesis-generating tools rather than definitive mechanistic proof. Docking analyses indicated that members of the AZTDS series are structurally capable of engaging two validated mycobacterial targets, DHFR and DprE1, through chemically reasonable interaction patterns. AZTDS-1, in particular, exhibited balanced binding profiles against both targets, consistent with its comparatively improved *in vitro* activity. MD simulations further refined these observations by demonstrating that AZTDS-1 forms stable complexes with both DHFR and DprE1 over extended timescales. While binding to DHFR appeared more flexible and adaptive, the interaction network with DprE1 was more persistent, characterized by sustained hydrogen bonding and hydrophobic contacts within the catalytic cavity. Post-MD MM-GBSA analysis supported these findings, highlighting dominant van der Waals, lipophilic, and electrostatic contributions to binding stability. Importantly, these computational results are interpreted as qualitative support for binding compatibility rather than quantitative predictors of cellular potency.

Several limitations of the present study should be acknowledged. First, the absence of direct biochemical inhibition assays prevents definitive assignment of DHFR or DprE1 as primary intracellular targets. Second, the moderate antimycobacterial potency indicates that further optimization will be required to improve cell wall penetration, intracellular accumulation, or target engagement. Finally, the reliance on *in silico* toxicity predictions, while informative at an early stage, requires experimental validation. Despite these limitations, this work introduces a chemically tractable thiadiazole–azetidinone hybrid scaffold with reproducible antibacterial and antimycobacterial activity, supported by coherent structure-based computational analyses. Rather than asserting target-specific inhibition, the study defines a rational starting point for subsequent medicinal chemistry efforts aimed at enhancing potency, selectivity, and pharmacokinetic properties. Collectively, the integrated experimental and computational results provide a solid foundation for further structure-guided optimization of this scaffold in antimicrobial drug discovery.

## 4. Material and methods

### 4.1 Chemical synthesis and characterization

All reagents and solvents were of analytical grade and used as received without further purification. Melting points were determined using open glass capillaries and are reported uncorrected. Reaction progress was monitored by thin-layer chromatography (TLC) on silica gel 60 F_254_ plates, using appropriate mixtures of ethyl acetate–chloroform or chloroform–methanol as mobile phases. TLC plates were visualized under UV light (254 nm) or by exposure to iodine vapors. Fourier-transform infrared (FTIR) spectra were recorded in the range of 4000–400 cm^−1^ using a Shimadzu FTIR spectrophotometer. ^1^H and ^13^C NMR spectra were recorded on a Bruker 400 MHz spectrometer, using tetramethylsilane as an internal standard. Chemical shifts (*δ*) are reported in parts per million (ppm), and spectra were acquired in DMSO-*d*_6_ or CDCl_3_ at ambient temperature. The amino-1,3,4-thiadiazole-2-sulfonamide intermediate (5-amino-1,3,4-thiadiazole-2-sulfonamide) is referred to as ATS, and the final thiadiazole–azetidinone hybrid derivatives are collectively designated as AZTDS throughout this work.

### 4.2 Synthesis of target compounds

#### 4.2.1 General procedure for preparation of ATS

**Scheme 1:**
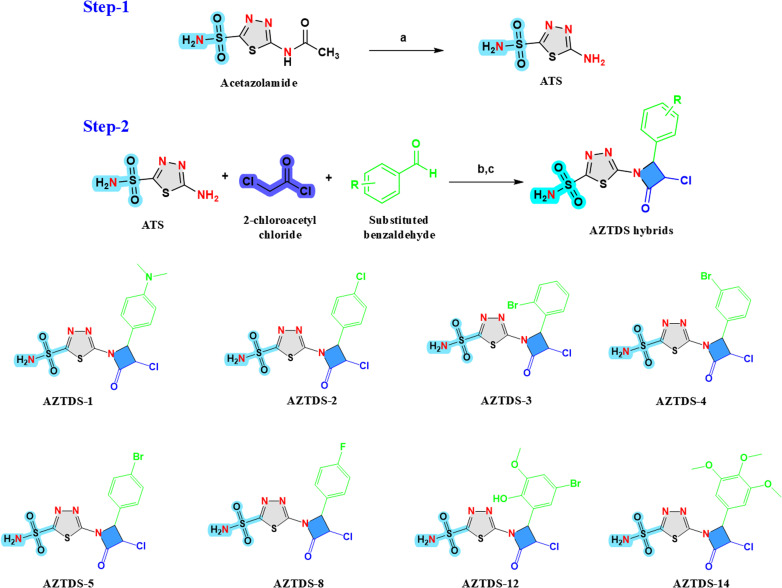
Synthesis of the AZTDS series compounds. Reagents and conditions: (a) concentrated HCl, reflux, 5 h; (b) ethanol; (c) substituted aromatic aldehydes (R–CHO), DMF/1,4-dioxane/ethanol, lithium hydroxide, stirred at room temperature for 6 h, chloro acetyl chloride, triethylamine, 3 h reflux. The structures of the substituted aromatic aldehydes (R) employed in the synthesis are depicted within the scheme.

The intermediate ATS was prepared starting from acetazolamide following an acid-mediated transformation. Acetazolamide (0.1 mol) was dissolved in concentrated hydrochloric acid (100 mL) and the reaction mixture was refluxed for 5 h.^32^ After completion of the reaction, the mixture was allowed to cool to room temperature and stirred for an additional 10 min. The resulting hydrochloride salt of ATS precipitated as a white solid and was collected by filtration. The crude hydrochloride salt was subsequently dissolved in distilled water (50 mL) and neutralized by the gradual addition of aqueous sodium bicarbonate until pH 7 was reached, leading to the precipitation of the free base. The solid product was filtered, washed with water, and crude compound was dissolved in suitable volume of methanol and heated to boiling. The solution was then kept overnight with mild stirring to promote slow recrystallization. The resulting crystals were filtered, washed with cold methanol to remove impurities, and dried to afford ATS as a white solid in 69% yield (Scheme 1).

#### 4.2.2 General procedure for the synthesis of AZTDS derivatives

5-Amino-1,3,4-thiadiazole-2-sulfonamide (ATS, 2.7 mmol, 1.0 equiv.) and the appropriate substituted aromatic aldehyde (2.7 mmol, 1.0 equiv.) were dissolved in absolute ethanol (10 mL). Lithium hydroxide (5.0 mmol, 1.8 equiv.) was added as a base, and the reaction mixture was refluxed for 5–6 h in the presence of activated 4 Å molecular sieves to facilitate dehydration and formation of the corresponding imine intermediate. The progress of imine formation was monitored by thin-layer chromatography (petroleum ether/ethyl acetate, 8 : 2).

After completion of the condensation step, the reaction mixture was allowed to cool to room temperature, and chloroacetyl chloride (2.7 mmol, 1.0 equiv.) was added dropwise, followed by the addition of triethylamine (1.0 equiv.) to neutralize the generated hydrochloric acid. The mixture was subsequently refluxed for an additional 3 h, promoting cyclization and formation of the azetidinone (β-lactam) ring. Upon completion of the reaction, the mixture was quenched by pouring into ice-cold water, and the resulting products were extracted with ethyl acetate/chloroform. The combined organic layers were concentrated under reduced pressure, and the crude products were purified by recrystallization from a methanol–water mixture to afford the desired AZTDS derivatives with good to moderate yields (52–72%).

### 4.3 Structural analysis of the compounds

The synthesized compounds were characterized by melting point determination, FTIR spectroscopy, and ^1^H NMR spectroscopy (SI S1). The spectroscopic data obtained are consistent with the proposed structures.

#### 4.3.1 ATS, 5-amino-1,3,4-thiadiazole-2-sulfonamide

Chemical formula: C_2_H_4_N_4_O_2_S_2_; white solid; yield: 69%; mp: 152–158 °C; ^1^H NMR (400 MHz, DMSO) *δ* 2.50–2.51 (1H, dd), 7.82 (1H, dd), 8.09 (1H, dd). ^13^C NMR (75 MHz, DMSO-*d*_6_) *δ* = 128.01, 138.83, 167.17, 170.24.

#### 4.3.2 AZTDS-1, 5-(3-chloro-2-(4-(dimethylamino)phenyl)-4-oxoazetidin-1-yl)-1,3,4-thiadiazole-2-sulfonamide

Chemical formula: C_13_H_14_ClN_5_O_3_S_2_; pale yellow solid; yield: 87.2%; mp: 79–81 °C; IR (KBr, cm^−1^): 3318.26 (N–H); 1374.26 (Ar–NH); 2919.42, 2824.25 (CH_3_); 1436.39 (S=O); 1238.38 (C–N); 1665.30 (C=O); 1315.07 (N–CH_3_); 1593.05 (N–H); ^1^H NMR (400 MHz, CDCl_3_) *δ* ppm = 3.96 (6H, s), 5.26 (1H, d), 7.15 (2H, ddd), 7.28 (2H, ddd), 9.89 (1H, d). ^13^C NMR (75 MHz, DMSO-*d*_6_) *δ* = 15.26, 39.72, 40.2, 48.29, 127.62, 136.21, 138.97, 149.18, 167.95, 170.09; HRMS (*m*/*z*): 388.24 [M + 1]^+^

#### 4.3.3 AZTDS-2, 5-(3-chloro-2-oxo-4-(*p*-tolyl)azetidin-1-yl)-1,3,4-thiadiazole-2-sulfonamide

Chemical formula: C_12_H_11_ClN_4_O_3_S_2_; white crystalline solid; yield: 77%; mp: 83–85 °C; IR (KBr, cm^−1^): 3314.05 (N–H); 1351.03 (Ar–NH); 2906.69 (CH_3_); 1440.72 (S=O); 1176.16 (C–N); 1606.08 (C=O); 1567 (N–H), 650.62 (CH); ^1^H NMR (400 MHz, DMSO) *δ* 2.22–2.23 (2H, s), 5.19–5.24 (3H, dd), 7.06–7.08 (2H, d), 7.43 (1H, ddd), 8.04 (1H, ddd).

#### 4.3.4 AZTDS-3, 5-(2-(2-bromophenyl)-3-chloro-4-oxoazetidin-1-yl)-1,3,4-thiadiazole-2-sulfonamide

Chemical formula: C_11_H_8_BrClN_4_O_3_S_2_; white crystalline solid; yield: 62.4%; mp: 145–149 °C; IR (KBr, cm^−1^): 3316.22 (N–H); 1374.26 (Ar–NH); 2963.47, 2923.96, 2824.25 (CH_3_); 1440.07 (S=O); 1268.67 (C–N); 1696.85.30 (C=O); 1496.40 (N–H); 649.72 (C–Br). ^1^H NMR (400 MHz, DMSO) *δ* 5.04 (1H, d), 5.48–5.50 (1H, d), 7.283 (2H, ddd), 7.289 (2H, ddd), 7.77 (1H, dd).

#### 4.3.5 AZTDS-4, 5-(2-(3-bromophenyl)-3-chloro-4-oxoazetidin-1-yl)-1,3,4-thiadiazole-2-sulfonamide

Chemical formula: C_11_H_8_BrClN_4_O_3_S_2_; off white solid; yield: 49.8%; mp: 142–148 °C; IR (KBr, cm^−1^): 3314.59 (N–H); 1350.37 (Ar–NH); 2926.34 (CH_3_); 1440.48 (S=O); 1175.91 (C–N); 1606.19 (C=O); 1497.25 (N–H); 649.92 (C–Br). ^1^H NMR (400 MHz, DMSO): *δ* 5.72–5.29 (1H, d), 5.69 (1H, d), 7.28–7.36 (3H, ddd), 7.49 (1H, dd).

#### 4.3.6 AZTDS-5, 5-(2-(4-bromophenyl)-3-chloro-4-oxoazetidin-1-yl)-1,3,4-thiadiazole-2-sulfonamide

Chemical formula: C_11_H_8_BrClN_4_O_3_S_2_; yellow crystalline solid; yield: 91.4%; mp: 144–148 °C; IR (KBr, cm^−1^): 3315.25 (N–H); 1351.81 (Ar–NH); 2931.13, 2857.30, 2813.21 (CH_3_); 1441.02 (S=O); 1283.71 (C–N); 1684.34 (C=O); 1584.34 (N–H), 660.06 (C–Br), ^1^H NMR (400 MHz, CDCl_3_): *δ* 5.29 (1H, d), 5.66–5.69 (1H, d), 7.26 (2H, ddd) 7.58 (1H, dd), 7.86–7.88 (2H, dd).

#### 4.3.7 AZTDS-8, 5-(3-chloro-2-(4-fluorophenyl)-4-oxoazetidin-1-yl)-1,3,4-thiadiazole-2-sulfonamide

Chemical formula: C_11_H_8_FClN_4_O_3_S_2_; orange color solid; yield: 52%; mp: 150–153 °C; IR (KBr, cm^−1^): 3316.27 (N–H); 1351.79 (Ar–NH); 2931.13, 2951.34 (CH_3_); 1440.27 (S=O); 1176.49 (C–N); 1497.29 (C=O); 1587.17 (N–H), 1084.06 (C–F), ^1^H NMR (400 MHz, DMSO): *δ* 5.21–5.24 (1H, d), 5.64 (1H, d), 7.06 (1H, dd), 7.64 (1H, ddd), 7.81 (1H, dd).

#### 4.3.8 AZTDS-12, 5-(2-(5-bromo-2-hydroxy-3-methoxyphenyl)-3-chloro-4-oxoazetidin-1-yl)-1,3,4-thiadiazole-2-sulfonamide

Chemical formula: C_12_H_10_BrClN_4_O_5_S_2_; white solid; yield: 56.4%; mp: 122–127 °C; IR (KBr, cm^−1^): 3315.31 (N–H); 1350.57 (Ar–NH); 2823.86 (CH_3_); 1444.64 (S=O); 1248.80 (C–N); 1662.20 (C=O); 1604.53 (N–H), 649.48 (C–Br), ^1^H NMR (400 MHz, MeOD): *δ* 3.73 (3H, s), 4.74 (1H, s), 6.46–6.49 (1H, d), 7.00–7.02 (2H, ddd) 7.08–7.13 (2H, dd), 7.64–7.70 (1H, d).

#### 4.3.9 AZTDS-14, 5-(3-chloro-2-oxo-4-(3,4,5-trimethoxyphenyl)azetidin-1-yl)-1,3,4-thiadiazole-2-sulfonamide

Chemical formula: C_14_H_15_ClN_4_O_6_S_2_; colourless needle shaped crystals; yield: 56.4%; mp: 65–68 °C; IR (KBr, cm^−1^): 3350.93 (N–H); 1390.55 (Ar–NH); 2946.68, 2842.94, 2801.97 (CH_3_); 1462.96 (S=O); 1237.69 (C–N); 1685.99 (C=O); 1588.09 (N–H), ^1^H NMR (400 MHz, CDCl_3_) *δ* 1.661 (2H, dd), 3.95–3.96 (9H, d), 7.15–7.28 (2H, dt), 9.89 (s, 1H).

### 4.4 Biological activity

#### 4.4.1 Antibacterial activity

The antibacterial activity of the synthesized compounds was evaluated *in vitro* against representative Gram-positive and Gram-negative bacterial strains using a broth-based serial dilution assay, as previously reported.^33^ The Gram-positive panel comprised *Staphylococcus aureus* (NCIM 5257) and *Bacillus subtilis* (NCIM 2097), while *Escherichia coli* (NCIM 2065) and *Pseudomonas aeruginosa* (NCIM 5210) were included as Gram-negative representatives. All strains were obtained from the National Collection of Industrial Microorganisms (NCIM), Pune, India. Assays were conducted in double-strength nutrient broth using standardized bacterial inocula prepared from freshly grown cultures, following established microbiological practices.^34^

Test compounds were dissolved in DMSO and evaluated over a concentration range of 1000–1.56 µg mL^−1^ using two-fold serial dilutions. Control experiments confirmed that the solvent concentrations employed did not affect bacterial growth. Amoxicillin was included as a reference antibacterial agent and tested under identical conditions. Following incubation at 37 °C for 24 h under aerobic conditions, antibacterial activity was assessed by visual inspection of culture turbidity. The minimum inhibitory concentration (MIC) was defined as the lowest concentration at which no visible bacterial growth was observed.

#### 4.4.2 Antimycobacterial activity

The antimycobacterial activity of selected compounds was assessed against *M. tuberculosis* H37Rv using the Microplate Alamar Blue Assay, following a reported protocol [XX]. Only compounds that demonstrated measurable antibacterial activity in the preliminary screening were advanced to antimycobacterial evaluation. All assays were performed in duplicate. Rifampicin and *p*-aminosalicylic acid were included as reference antitubercular agents. To minimize evaporation effects during prolonged incubation, the peripheral wells of sterile 96-well microplates were filled with sterile deionized water. The remaining wells were charged with Middlebrook 7H9 broth, and two-fold serial dilutions of the test compounds were prepared directly in the assay plate. A standardized *M. tuberculosis* H37Rv inoculum was then added to each well.

The plates were sealed and incubated at 37 °C for 5 days. Following incubation, a freshly prepared 1 : 1 mixture of Alamar Blue reagent and 10% Tween 80 was added to each well, and the plates were further incubated for 24 h at 37 °C. Mycobacterial growth was assessed by visual inspection of the colorimetric response of the indicator. The minimum inhibitory concentration was defined as the lowest concentration at which no color change from blue to pink was observed.

### 4.5 Molecular modelling studies

#### 4.5.1 Molecular docking

Molecular docking studies were performed using MzDOCK v1.0, a GUI-based open-source platform integrating an automated docking workflow.^35^ The crystal structures of *M. tuberculosis* DprE1 (PDB ID: 6G83) and DHFR (PDB ID: 4KLX) were retrieved from the RCSB Protein Data Bank and used as docking targets. Protein structures were prepared by removing crystallographic water molecules and heteroatoms, completing missing residues where necessary, and adding hydrogen atoms and Kollman charges.^36^ The stereochemical quality of the prepared protein structures was evaluated using Ramachandran plot analysis of chain A. Ligand structures were generated using ChemDraw (version 16.0.1.4), converted to three-dimensional geometries, and energy-minimized using the MMFF94 force field. Ligands were protonated to reflect physiological pH conditions (pH 7.4).

To validate the docking protocol, redocking of the co-crystallized ligands was performed, and the resulting poses were compared with the experimental binding conformations. The close agreement between the redocked and crystallographic poses confirmed the reliability of the docking setup. Docking calculations were conducted using the Smina-Vina scoring function, with the grid box centered on the co-crystallized ligand and extended by 4 Å in each dimension. The exhaustiveness and number of output poses were set to 9. Docked conformations were analyzed and visualized using Discovery Studio Visualizer, PyMOL, and ICM Browser Pro.^37,38^

#### 4.5.2 Molecular dynamics simulation

The most active antimycobacterial compound was further investigated by MD simulations to evaluate the stability and dynamic behavior of its complexes with DprE1 and DHFR. All MD simulations were performed using the Desmond module implemented in the Schrödinger Maestro 2024–2 suite, employing the OPLS4 force field. Each protein–ligand complex was solvated in an orthorhombic simulation box using the TIP3P water model, with a minimum buffer distance of 10 Å between the protein surface and the box boundaries. The systems were neutralized by the addition of appropriate counterions (Na^+^/Cl^−^) and adjusted to an ionic strength of 0.15 M NaCl. Energy minimization and equilibration were carried out using the default Desmond relaxation protocol prior to the production phase. Production MD simulations were conducted for 200 ns under NPT conditions, maintaining the temperature at 300 K and the pressure at 1 atm. A time step of 2 fs was applied throughout the simulations, with covalent bonds involving hydrogen atoms constrained using the SHAKE algorithm. Trajectories were analyzed to assess structural stability and interaction persistence, including root-mean-square deviation (RMSD), root-mean-square fluctuation (RMSF), ligand binding stability, and protein–ligand interaction profiles.^39^

Binding free energy calculations were performed using the Prime MM-GBSA module of the Schrödinger suite. MM-GBSA estimates were computed from representative protein–ligand complexes and incorporate contributions from bonded and non-bonded interactions as well as polar and non-polar solvation terms using the VSGB solvation model. The binding free energy (Δ*G*_binding) was calculated according to eqn (1).

1 Δ*G*_binding_ = *G*_complex_ − (*G*_protein_ + *G*_ligand_)

#### 4.5.3 *In silico* prediction of ADMET properties

*In silico* prediction of absorption, distribution, metabolism, excretion, and toxicity (ADMET) parameters were performed to obtain a preliminary assessment of the drug-likeness and safety profile of the synthesized compounds. Pharmacokinetic descriptors related to oral bioavailability and physicochemical behavior were calculated using the SwissADME web server (https://www.swissadme.ch/index.php). In parallel, toxicity-related endpoints, including acute toxicity risk classification and estimated toxicological liabilities, were evaluated using the ProTox-3.0 platform.^40,41^ These computational analyses were employed as supportive tools to contextualize the experimental findings and to identify potential liabilities at an early stage of compound optimization.

## Author contributions

Vishwakarma S. K.: writing – original draft, resources, methodology, investigation, conceptualization. P. Naresh: writing – review & editing, supervision, project administration. M. Achal: data visualization, validation; Roque-Borda C. A.: writing – review & editing, data curation, validation.

## Conflicts of interest

There are no conflicts of interest.

## Supplementary Material

RA-016-D6RA00735J-s001

## Data Availability

The data supporting the findings of this study are available within the article and its supplementary information (SI). Additional data are available from the corresponding author upon reasonable request. Supplementary information is available. See DOI: https://doi.org/10.1039/d6ra00735j.
