# Supplementary material for: Thiadiazole–azetidinone sulfonamide hybrids with antimycobacterial activity supported by structure-based analysis
Source: RSC Adv. 2026 Mar 19;16(17):15553–68. doi: 10.1039/d6ra00735j (PMC13000902; doi:10.1039/d6ra00735j)
Supplement: RA-016-D6RA00735J-s001 [file RA-016-D6RA00735J-s001.pdf]

Supplementary Information (SI) for RSC Advances.
This journal is © The Royal Society of Chemistry 2026

Supplementary Material

Thiadiazole–azetidinone sulfonamide hybrids with antimycobacterial activity supported by structure-based analysis

Subham Kumar Vishwakarma[1,2]; Achal Mishra[3]; Naresh Panigrahi[1*]; Cesar Augusto Roque-Bord[4*]

[1] GITAM School of Pharmacy, GITAM Deemed to be University, Rushikonda, Visakhapatnam-530045, (A.P.), India.

[2] School of Pharmaceutical Sciences, São Paulo State University (UNESP), 14800903, Araraquara, Brazil

[3] Department of Pharmacy, Guru Ghasidas Vishwavidyalaya, Bilaspur– 495009, (C.G.), India

[4] Vicerrectorado de Investigación, Universidad Católica de Santa María, Arequipa, Peru.

Correspondence

* Cesar Augusto Roque-Borda, Vicerrectorado de Investigación, Universidad Católica de Santa María, Arequipa, Peru. e-mail: cesar.roque@ucsm.edu.pe

* Naresh Panigrahi, GITAM School of Pharmacy, GITAM Deemed to be University, Rushikonda, Visakhapatnam-530045, (A.P.), India.

Description of the Spectral Analysis

1. Spectral data of ATS

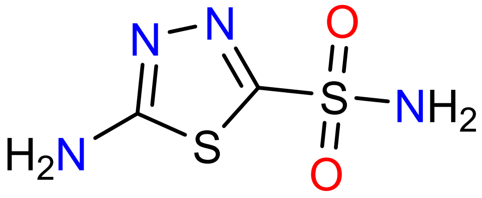

^{1}H NMR

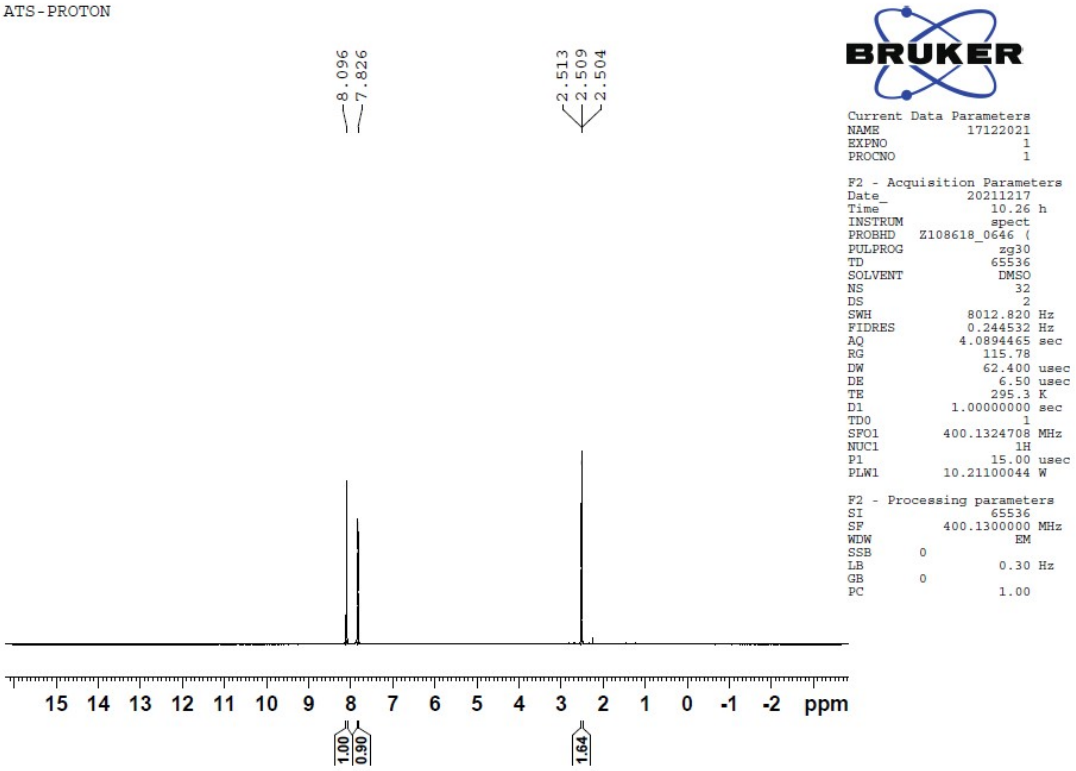

^{13}C NMR

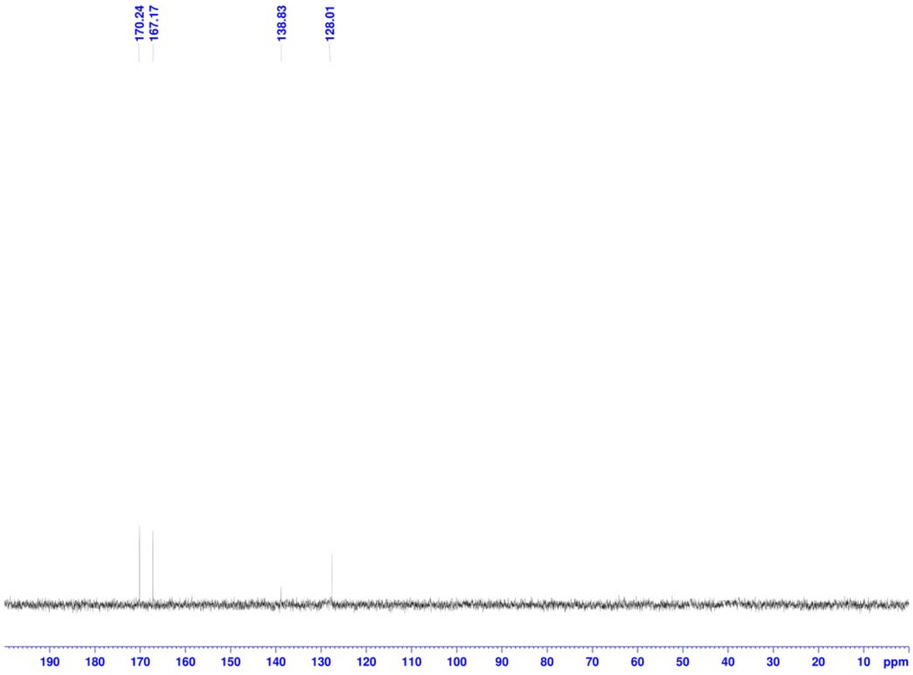

BRUKER

Current Data Parameters
NAME A-1
EXPNO 2
PROCNO 1

F2 - Acquisition Parameters
Date_ 20220206
Time 3.39
INSTRUM FOURIER300
PROBHD 5 mm DUL 13C-1
PULPROG zgpg
TD 32768
SOLVENT DMSO
NS 2048
DS 4
SWH 24414.062 Hz
FIDRES 0.745058 Hz
AQ 0.6710886 sec
RG 501.187
DW 20.480 usec
DE 6.50 usec
TE 298.2 K
D1 1.00000000 sec
D11 0.03000000 sec
D31 0.00001500 sec
D32 0.89999998 sec
D40 0.00093990 sec
L4 23
L5 26
P32 90.00 usec
TD0 1

======== CHANNEL f1 ========
SFO1 75.4878687 MHz
NUC1 13C
P1 15.00 usec
PLW1 15.00000000 W

======== CHANNEL f2 ========
SFO2 300.1812007 MHz
NUC2 1H
CPDPRG[2 waltz16
PCPD2 90.00 usec
PLW2 10.00000000 W
PLW12 0.20863999 W
PLW13 0.10495000 W

F2 - Processing parameters
SI 32768
SF 75.4803210 MHz
WDW EM
SSB 0
LB 1.00 Hz
GB 0
PC 1.40

2. Spectral data of AZTDS-1

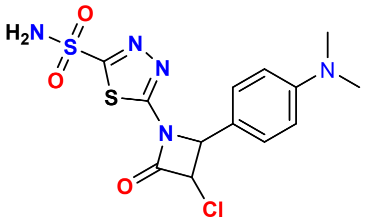

IR spectra

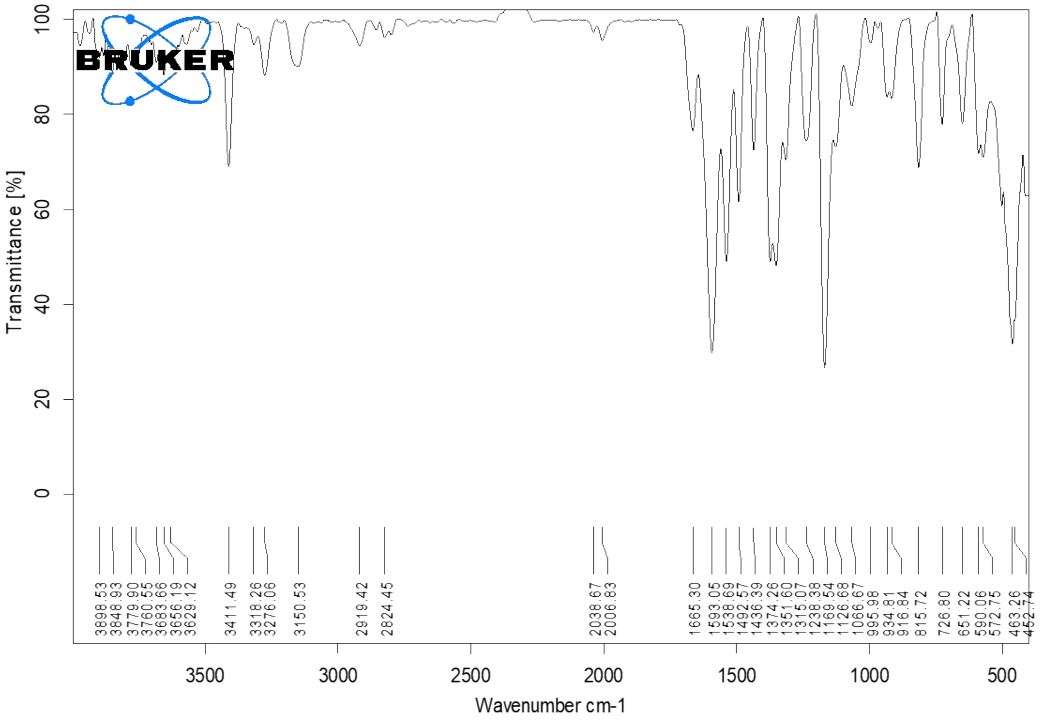

^{1}H NMR

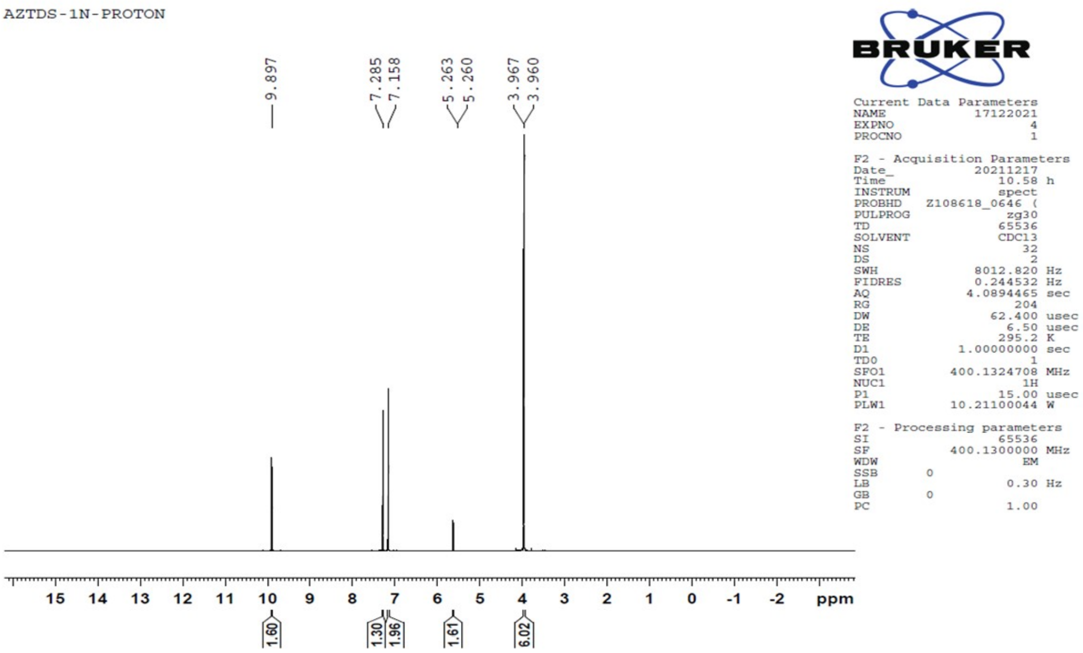

AZTDS-1N-PROTON
9.897
7.285
7.158
5.263
5.260
3.967
3.960
15 14 13 12 11 10 9 8 7 6 5 4 3 2 1 0 -1 -2 ppm
1.60
1.30
1.96
1.61
6.02
BRUKER
Current Data Parameters
NAME 17122021
EXPNO 4
PROCNO 1
F2 - Acquisition Parameters
Date_ 20211217
Time 10.58 h
INSTRUM spect
PROBHD Z108618_0646 (
PULPROG zg30
TD 65536
SOLVENT CDCl3
NS 32
DS 2
SWH 8012.820 Hz
FIDRES 0.244532 Hz
AQ 4.0894465 sec
RG 204
DW 62.400 usec
DE 6.50 usec
TE 295.2 K
D1 1.00000000 sec
TD0 1
SFO1 400.1324708 MHz
NUC1 1H
P1 15.00 usec
PLW1 10.21100044 W
F2 - Processing parameters
SI 65536
SF 400.1300000 MHz
WDW EM
SSB 0
LB 0.30 Hz
GB 0
PC 1.00

^{13}C NMR

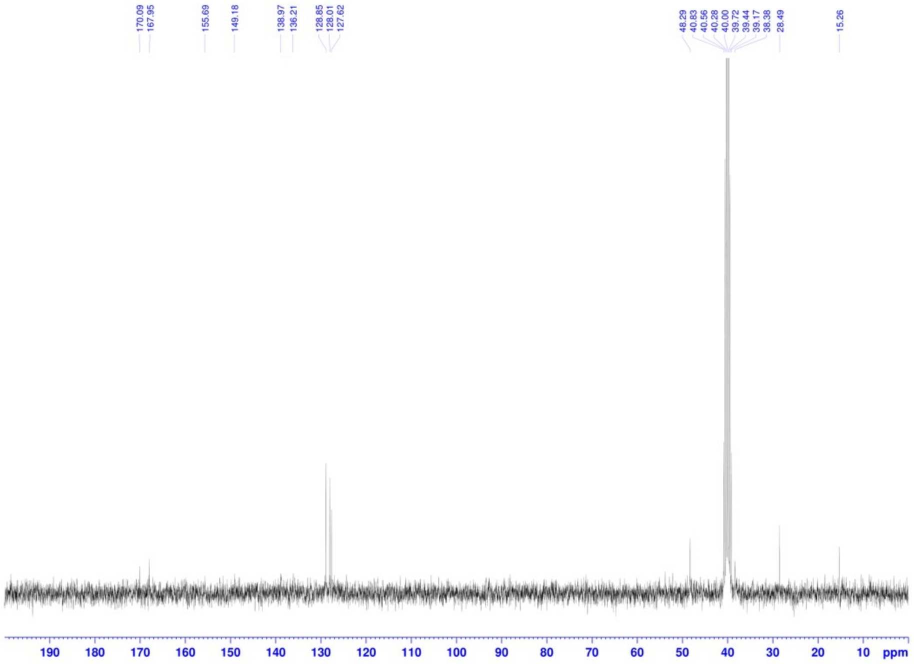

Current Data Parameters
NAME S-1
EXPNO 2
PROCNO 1

F2 - Acquisition Parameters
Date_ 20220206
Time 4.39
INSTRUM FOURIER300
PROBHD 5 mm DUL 13C-1
PULPROG zgpg
TD 32768
SOLVENT DMSO
NS 2048
DS 4
SWH 24414.062 Hz
FIDRES 0.745058 Hz
AQ 0.6710886 sec
RG 501.187
DW 20.480 usec
DE 6.50 usec
TE 298.2 K
D1 1.00000000 sec
D11 0.03000000 sec
D31 0.00001500 sec
D32 0.89999998 sec
D40 0.00093990 sec
L4 23
L5 26
P32 90.00 usec
TD0 1

======== CHANNEL f1 ========
SFO1 75.4878687 MHz
NUC1 13C
P1 15.00 usec
PLW1 15.00000000 W

======== CHANNEL f2 ========
SFO2 300.1812007 MHz
NUC2 1H
CPDPRG[2 waltz16
PCPD2 90.00 usec
PLW2 10.00000000 W
PLW12 0.20863999 W
PLW13 0.10495000 W

F2 - Processing parameters
SI 32768
SF 75.4803210 MHz
WDW EM
SSB 0
LB 1.00 Hz
GB 0
PC 1.40

HRMS

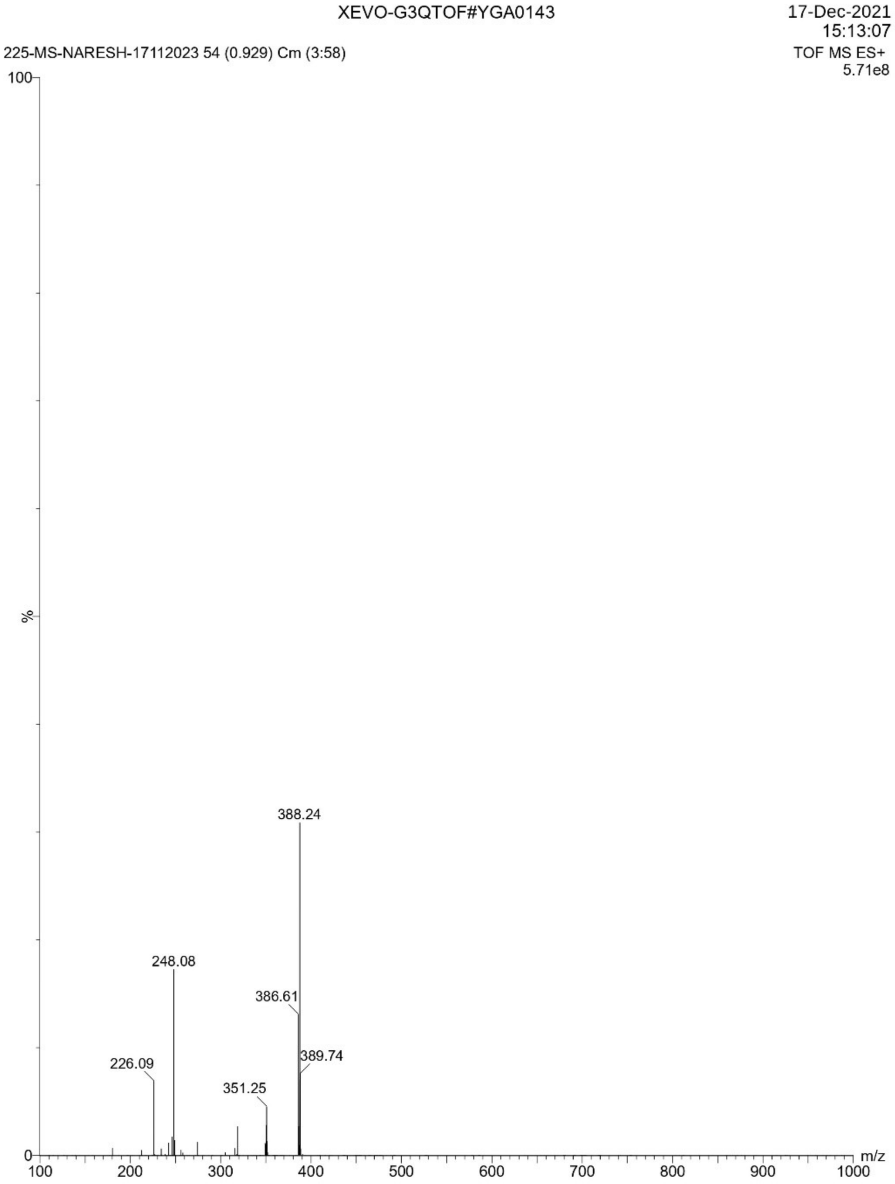

3. Spectral data of AZTDS-2

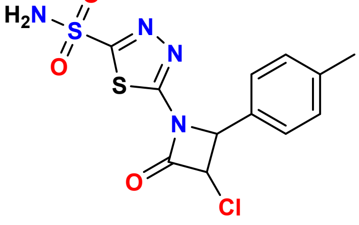

IR spectra

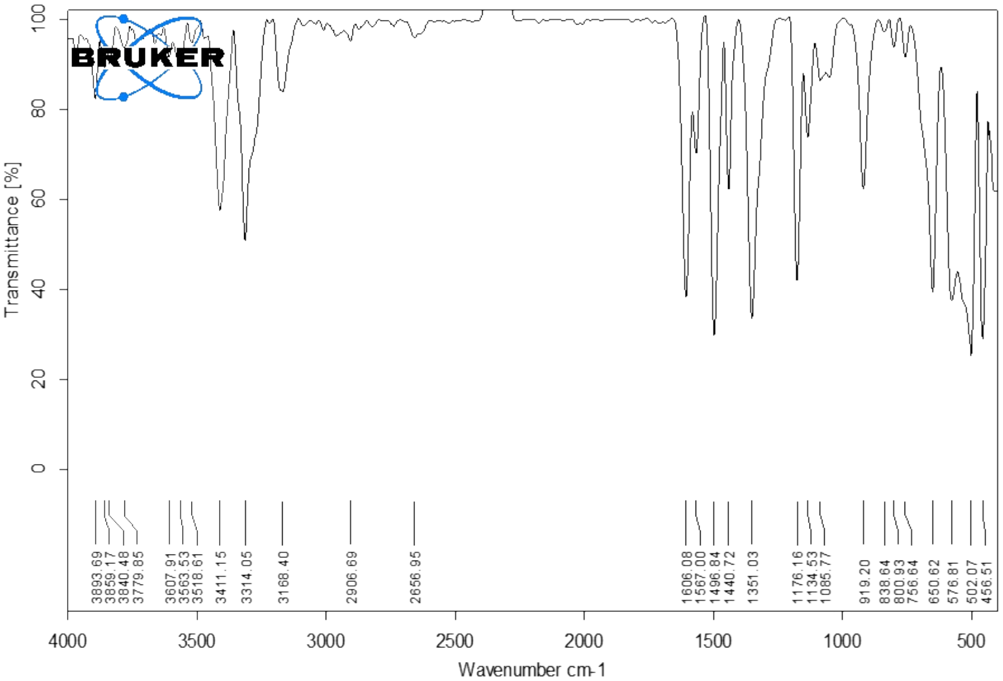

^{1}H NMR

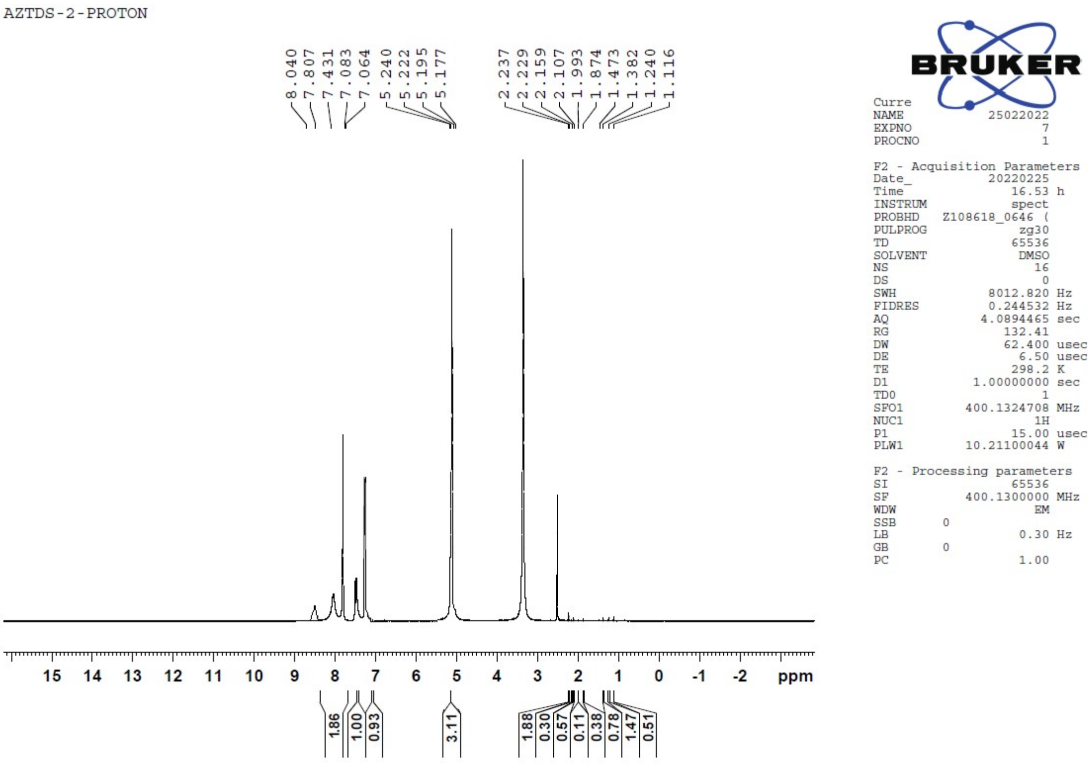

4. Spectral data of AZTDS-3

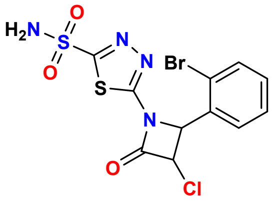

IR spectra

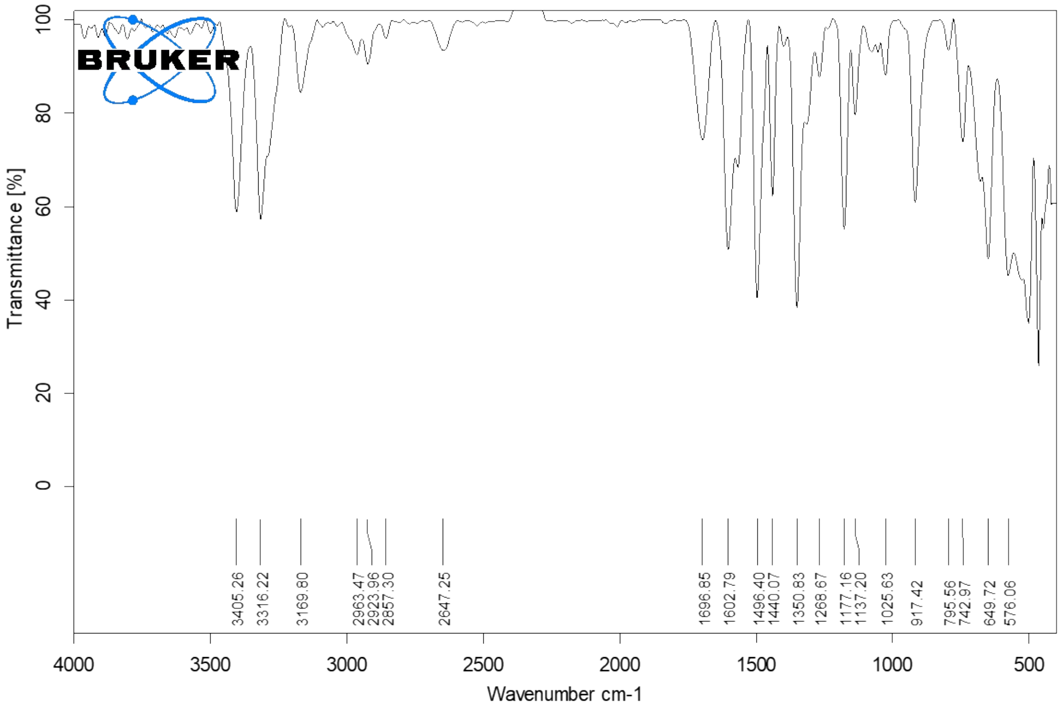

BRUKER
Transmittance [%]
Wavenumber cm-1
3405.26
3316.22
3169.80
2963.47
2923.96
2857.30
2647.25
1696.85
1602.79
1496.40
1440.07
1350.83
1268.67
1177.16
1137.20
1025.63
917.42
795.56
742.97
649.72
576.06

^{1}H NMR

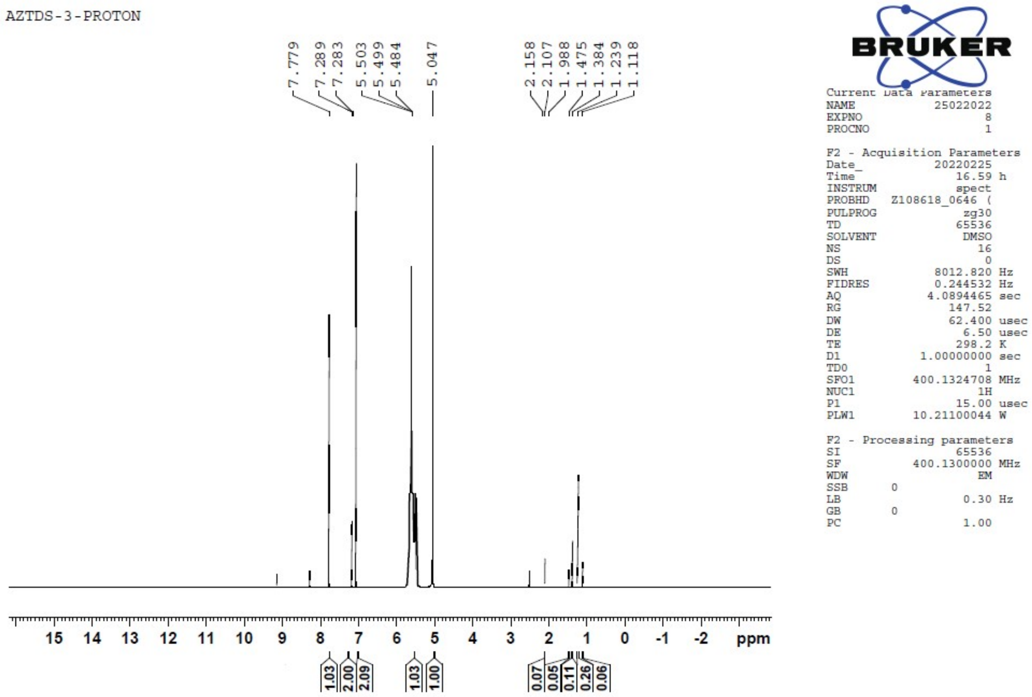

5. Spectral data of AZTDS-4

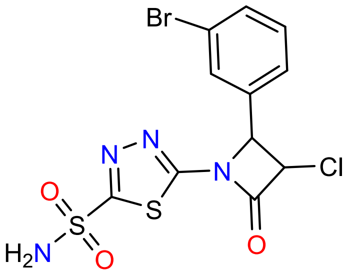

IR Spectra

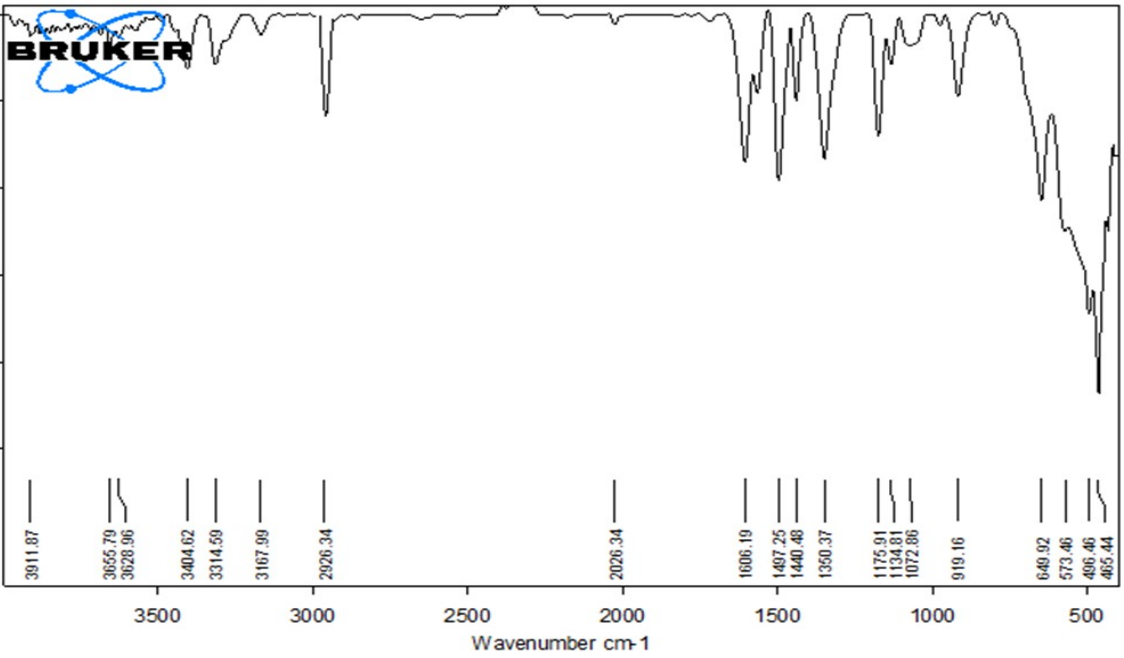
BRUKER
3911.87
3655.79
3628.96
3404.62
3314.59
3167.99
2926.34
2026.34
1606.19
1497.25
1440.48
1350.37
1175.91
1134.81
1072.86
919.16
649.92
573.46
496.46
465.44
3500
3000
2500
2000
1500
1000
500
Wavenumber cm-1

^{1}H NMR

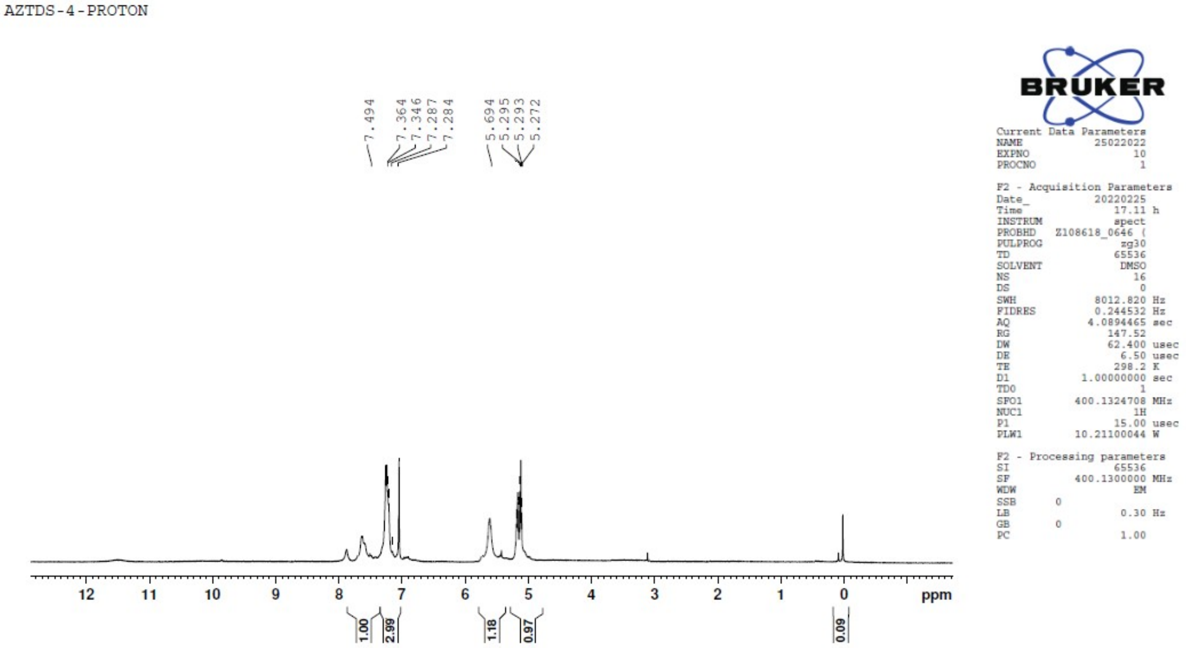

6. Spectral data of AZTDS-5

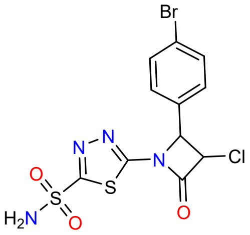

IR spectra

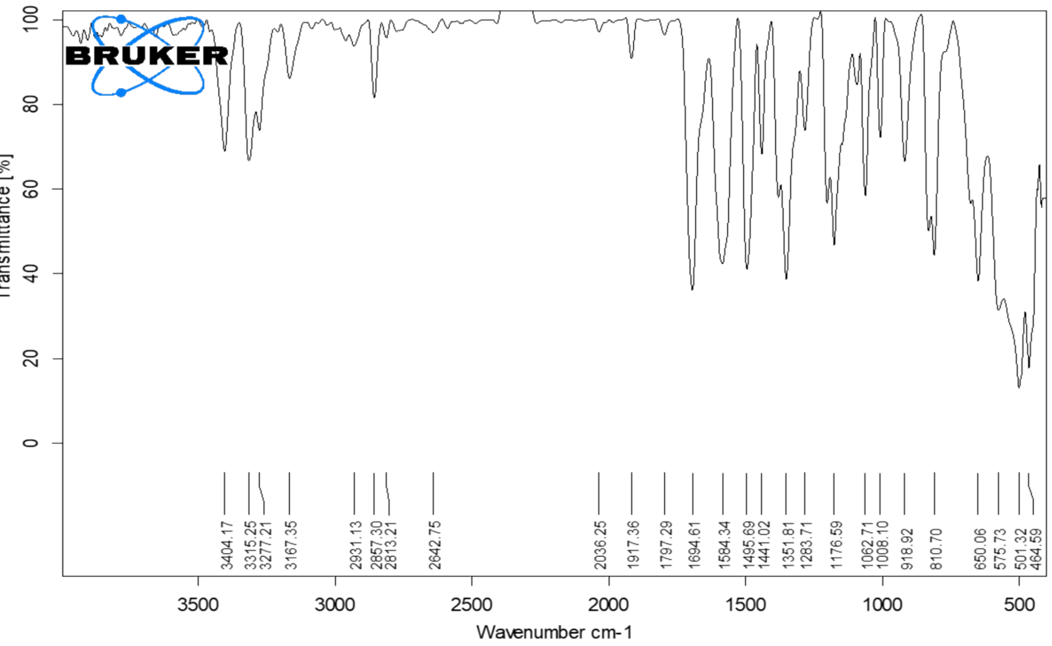
BRUKER
Transmittance [%]
100
80
60
40
20
0
3404.17
3315.25
3277.21
3167.35
2931.13
2857.30
2813.21
2642.75
2036.25
1917.36
1797.29
1694.61
1584.34
1495.69
1441.02
1351.81
1283.71
1176.59
1062.71
1008.10
918.92
810.70
650.06
575.73
501.32
464.59
3500
3000
2500
2000
1500
1000
500
Wavenumber cm-1

^{1}H NMR

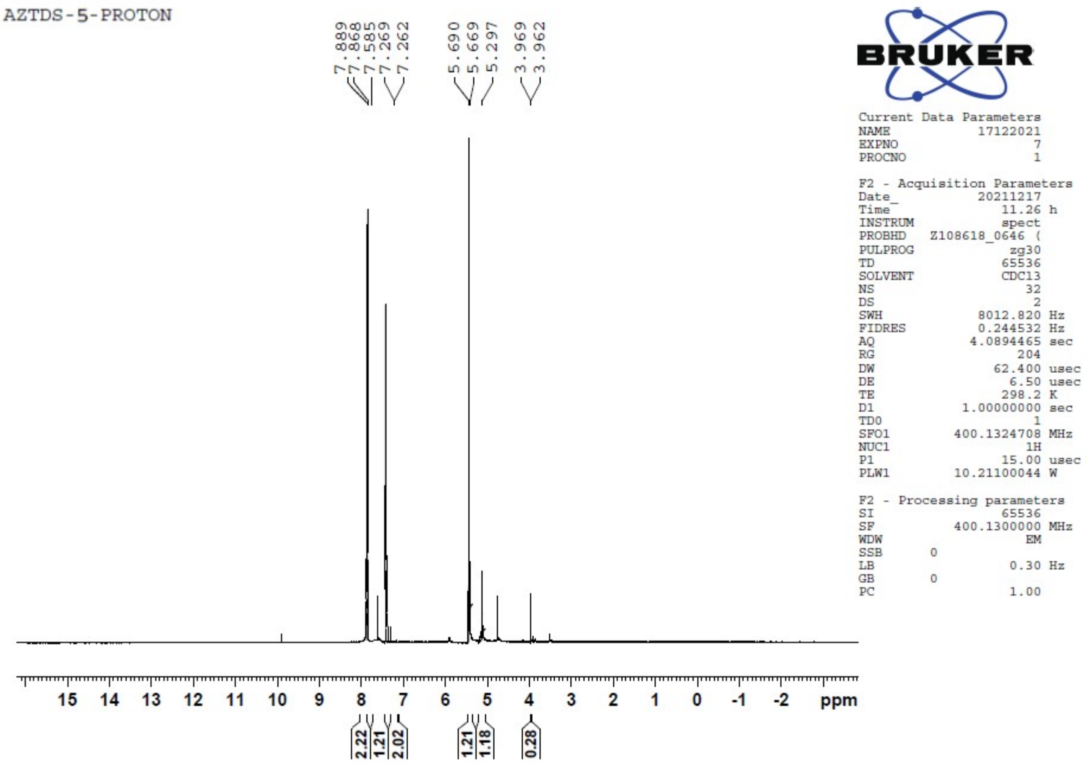

7. Spectral data of AZTDS-8

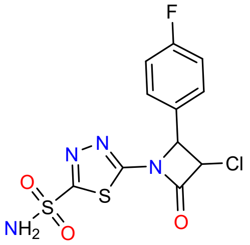

IR Spectra

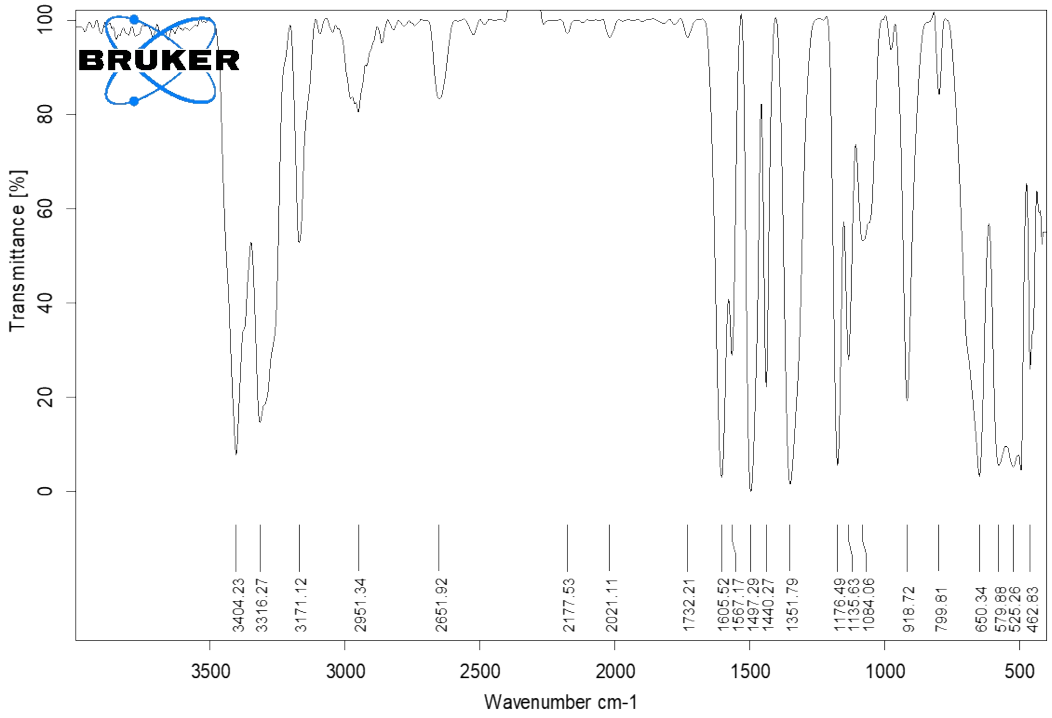
BRUKER
Transmittance [%]
100
80
60
40
20
0
3404.23
3316.27
3171.12
2951.34
2651.92
2177.53
2021.11
1732.21
1605.52
1567.17
1497.29
1440.27
1351.79
1176.49
1135.63
1084.06
918.72
799.81
650.34
579.88
525.26
462.83
3500
3000
2500
2000
1500
1000
500
Wavenumber cm-1

^{1}H NMR

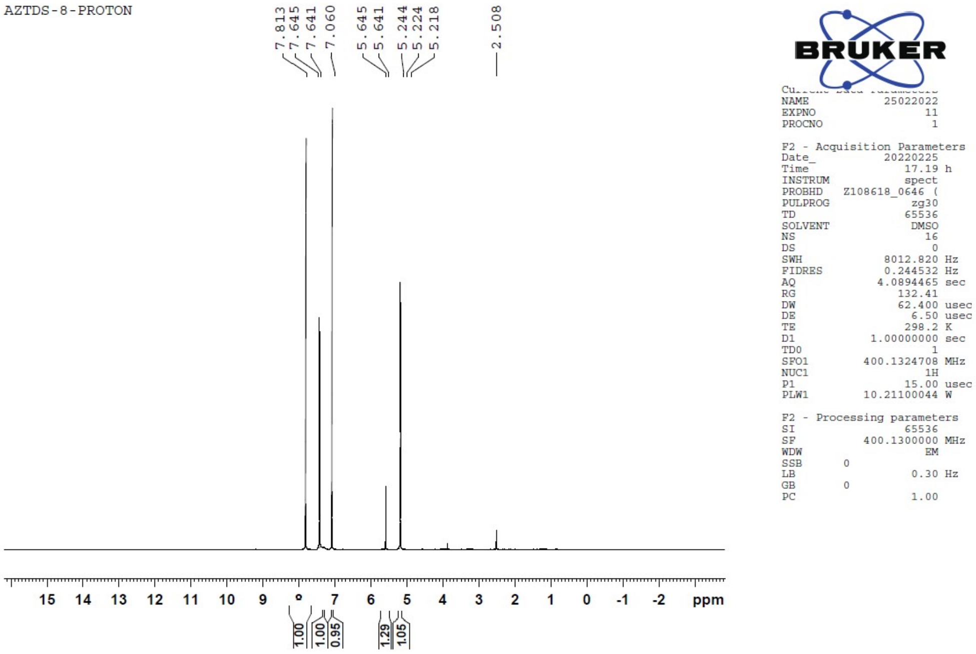

8. Spectral data of AZTDS-12

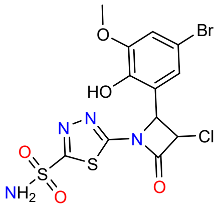

IR spectra

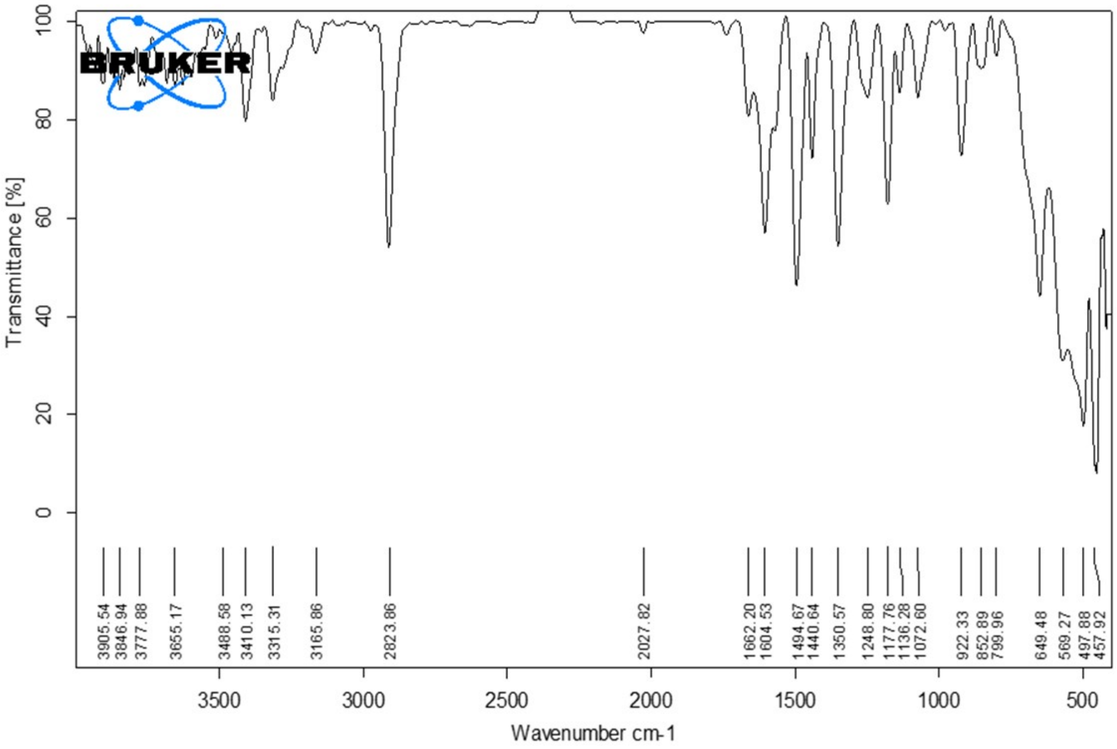
BRUKER
Transmittance [%]
100
80
60
40
20
0
3905.54
3846.94
3777.88
3655.17
3488.58
3410.13
3315.31
3165.86
2823.86
2027.82
1662.20
1604.53
1494.67
1440.64
1350.57
1248.80
1177.76
1136.28
1072.60
922.33
852.89
799.96
649.48
569.27
497.88
457.92
3500
3000
2500
2000
1500
1000
500
Wavenumber cm-1

^{1}H NMR

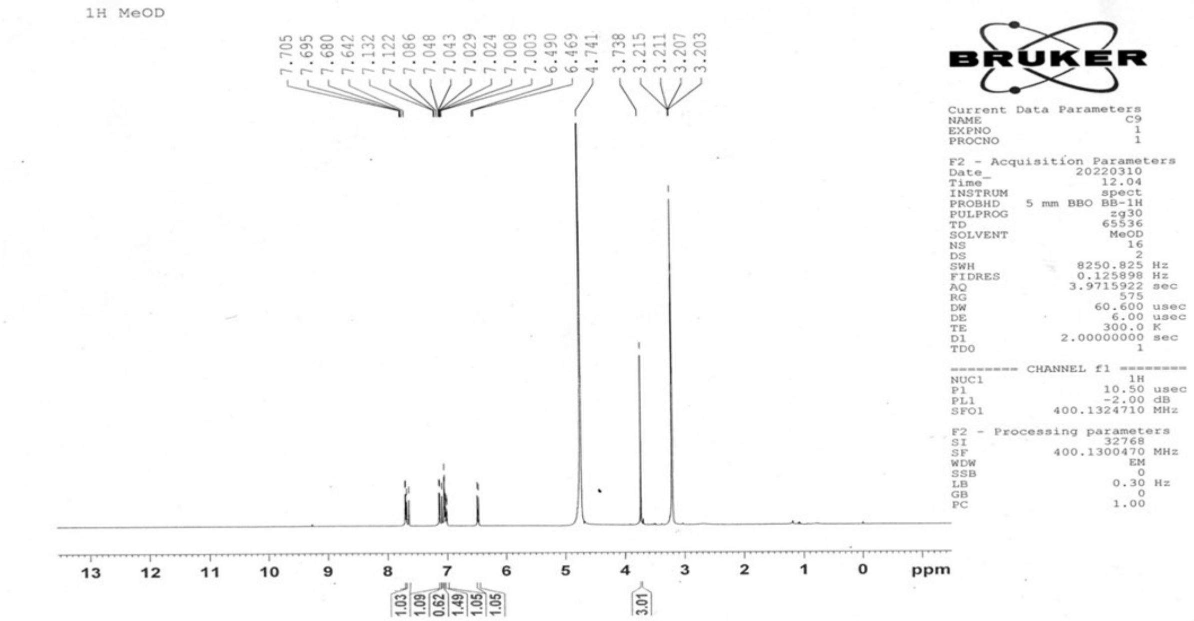

9. Spectral data of AZTDS-14

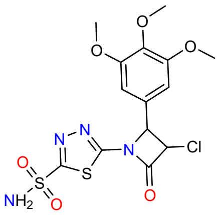

IR spectra

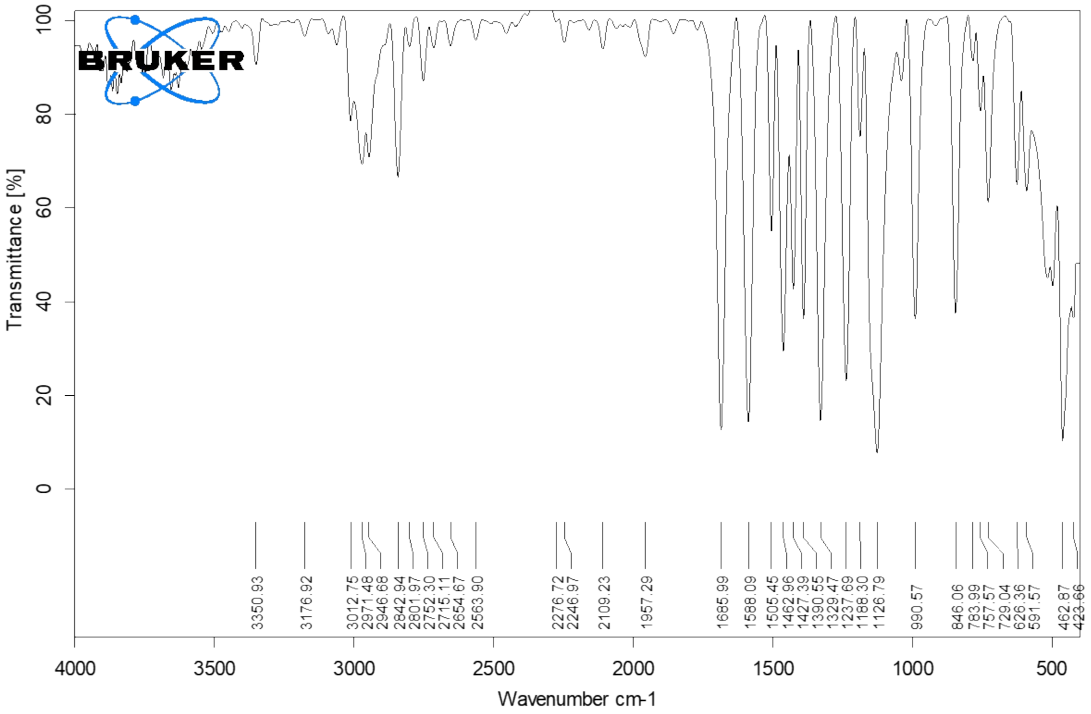
Transmittance [%]
Wavenumber cm-1
BRUKER
3350.93
3176.92
3012.75
2971.48
2946.68
2842.94
2801.97
2752.30
2715.11
2654.67
2563.90
2276.72
2246.97
2109.23
1957.29
1685.99
1588.09
1505.45
1462.96
1427.39
1390.55
1329.47
1237.69
1188.30
1126.79
990.57
846.06
783.99
757.57
729.04
626.36
591.57
462.87
423.66

^{1}H NMR

AZTDS-14-PROTON

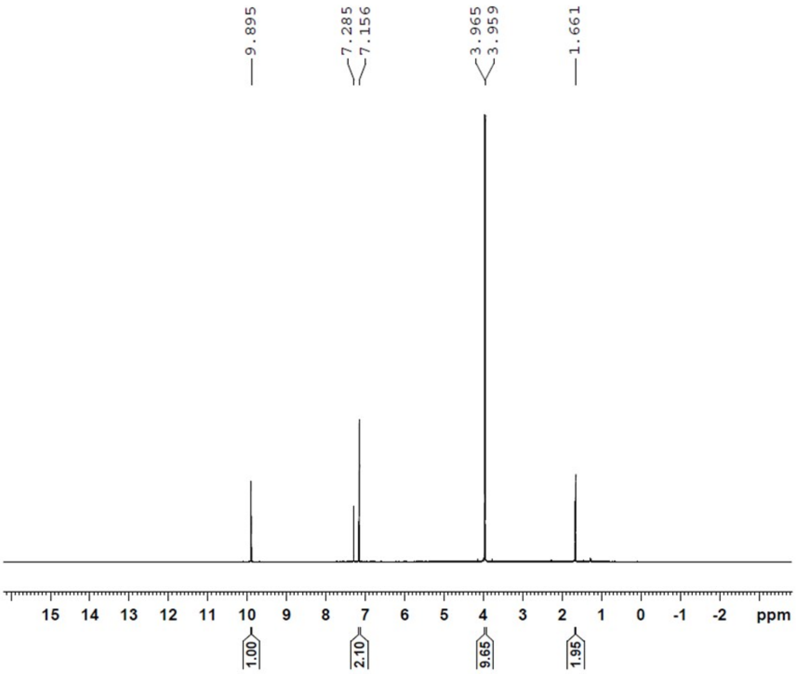

Current Data Parameters

```
NAME            18022022
EXPNO                  1
PROCNO                 1

F2 - Acquisition Parameters
Date_           20220218
Time               11.59 h
INSTRUM            spect
PROBHD   Z108618_0646 (
PULPROG             zg30
TD                 65536
SOLVENT            CDCl3
NS                    32
DS                     2
SWH             8012.820 Hz
FIDRES          0.244532 Hz
AQ             4.0894465 sec
RG                162.09
DW                62.400 usec
DE                  6.50 usec
TE                 293.4 K
D1            2.00000000 sec
TD0                    1
SFO1         400.1324708 MHz
NUC1                  1H
P1                 15.00 usec
PLW1         10.21100044 W

F2 - Processing parameters
SI                 65536
SF           400.1300000 MHz
WDW                   EM
SSB       0
LB                  0.30 Hz
GB        0
PC                  1.00
```
